# On the processes influencing rapid intensity changes of tropical cyclones over the Bay of Bengal

**DOI:** 10.1038/s41598-019-40332-z

**Published:** 2019-03-04

**Authors:** Saiprasanth Bhalachandran, R. Nadimpalli, K. K. Osuri, F. D. Marks Jr., S. Gopalakrishnan, S. Subramanian, U. C. Mohanty, D. Niyogi

**Affiliations:** 1 Purdue University, Department of Earth, Atmospheric and Planetary Sciences, West Lafayette, 47906 USA; 2 Indian Institute of Technology Bhubaneshwar, Bhubaneshwar, India; 3 National Institute of Technology, Rourkela, India; 4 NOAA Hurricane Research Division, Miami, FL USA; 5 Department of Agronomy, Purdue University, West Lafayette, 47906 USA

## Abstract

We present a numerical investigation of the processes that influenced the contrasting rapid intensity changes in Tropical Cyclones (TC) Phailin and Lehar (2013) over the Bay of Bengal. Our emphasis is on the significant differences in the environments experienced by the TCs within a few weeks and the consequent differences in their organization of vortex-scale convection that resulted in their different rapid intensity changes. The storm-relative proximity, intensity, and depth of the subtropical ridge resulted in the establishment of a low-sheared environment for Phailin and a high-sheared environment for Lehar. Our primary finding here is that in Lehar’s sheared vortex, the juxtaposition in the azimuthal phasing of the asymmetrically distributed downward eddy flux of moist-entropy through the top of the boundary layer, and the radial eddy flux of moist-entropy within the boundary layer in the upshear left-quadrant of Lehar (40–80 km radius) establishes a pathway for the low moist-entropy air to intrude into the vortex from the environment. Conversely, when the azimuthal variations in boundary layer moist-entropy, inflow, and convection are weak in Phailin’s low-sheared environment, the inflow magnitude and radial location of boundary layer convergence relative to the radius of maximum wind dictated the rapid intensification.

## Introduction

During the early hours of 10 October 2013, Tropical Cyclone (TC) Phailin began rapidly intensifying across the Bay of Bengal (BoB). Over the next 24 hours, there was an increase in maximum surface wind speed from 45 knots to 115 knots (23.15 m/s to 59.1 m/s) and a central pressure drop from 996 hPa to 940 hPa. Phailin’s rapid deepening presented a major challenge to the response teams as a massive evacuation of approximately a million people had to be coordinated^1^. Only six weeks later, TC Lehar reached an intensity of 75 knots (38.5 m/s, Very Severe Cyclonic Status) and concerns similar to Phailin were raised as the storm approached land. However, over the next 18 hours, Lehar went on to weaken rapidly to 30 knots, well before landfall. Such contrasting rapid changes in TC intensity (RIC) are a nightmare to forecasters and policymakers. The BoB is a relatively small basin, and when such massive evacuations are at stake, ‘erring on the safe side’ is not a welcome option since false alarms tend to reduce the credibility of the forecasts and subsequent response from the public. Early and accurate forecasts and warnings can help prepare disaster response teams in advance and save thousands of lives and property, and every step towards an improved fundamental understanding of the mechanisms driving these intensity changes goes a long way in ensuring the same.

A change in a TC’s intensity is the result of various dynamic and thermodynamic processes interacting at and across multiple scales. These processes range from the large-scale circulations in the TC atmospheric environment, the mesoscale organized convective processes at the vortex-scale, down to the sub-grid scale microphysical processes^2–6^. As a result, there are multiple pathways possible for a TC vortex that lead it to rapid intensification (RI, defined as an increase of at least 30 knots over a period of 24 hours^7^) or rapid weakening (RW, a decrease of 30 knots or more over a period of 24 hours^8^). Prior research has suggested that there are no *special* set of processes that govern RICs that are different from the processes that drive the intensity changes of TCs that do not undergo RICs^9^. Given a reasonably favorable environment and a vortex structure, all TCs are expected to intensify unless they are subject to vertical wind shear (hereafter ‘shear’), dry air or relatively low sea surface temperatures^7,9–11^.

However, when a TC is subject to shear, the problem of diagnosing RICs becomes more complicated^12,13^. The structure of a sheared vortex deviates significantly from its azimuthally-averaged structures, primarily due to the dominance of wavenumber-1 (and higher order) asymmetries. What we understand from past numerical^14,15^ and observational examinations^11,16^ of sheared vortices is that when the vortex tilts, a series of thermal and vertical motion anomalies occur to restore thermal wind balance (upward motion and cold anomaly downtilt, and downward motion and warm anomaly uptilt). In essence, shear establishes the preferred azimuthal location of convection such that the updrafts are predominant in the downshear quadrants and the downdrafts are dominant in the upshear quadrants^15,17–19^. In addition to shear, the storm motion magnitude and direction^20,21^, as well as air-sea temperature and moisture disequilibrium, may also act simultaneously to influence the radial and azimuthal distribution of convection^22–25^. Therefore, it is insufficient to conclude a diagnostic analysis of a TC by classifying it merely as a “sheared” storm or a non-sheared storm. This is because, while TCs have traditionally known to weaken in a sheared-environment, recent studies^15,26–30^ have articulated different possibilities of how TCs can intensify in a sheared environment. Furthermore, with constant feedback from the vortex to the environment, the environment also evolves in parallel with the TC vortex. Under such a scenario, the surrounding moist entropy, vortex resiliency, depth of the vortex, stage of the storm, storm motion vector, and the vertical structure of shear, all help establish the context in which shear interacts with the vortex, and that ultimately determines the fate of the storm.

Previous studies focused on the processes responsible for the weakening of TCs in a sheared environment^31–35^ have primarily relied on idealized numerical experiments and theoretical expositions to diagnose the mechanisms responsible for TC weakening. A recurrent theme amongst all these studies, is the presence of low moist-entropy (or equivalent potential temperature, *θ*_*e*_) air in the TC environment, and a shear-induced pathway for this low *θ*_*e*_ air to intrude into the storm vortex, after which, the low *θ*_*e*_ air acts as the storm’s anti-fuel and the ability to sustain deep convection is lost. However, the environmental air cannot penetrate or influence the TC inner core unless the TC-relative flow is sufficiently strong or the vortex is weak^32^. Tang and Emanuel^33,34^ using an axisymmetric model, concluded that inward eddy moist-entropy fluxes at mid-levels (through vortex-Rossby waves excited by shear-vortex interactions), were responsible for the ventilation of the TC-inner core with environmental air, and subsequent weakening of the TCs. On the other hand, Riemer and Laliberte^35^ using a trajectory analysis in an asymmetric framework, affirmed the conclusion of Riemer *et al*.^31^ that the dominant pathway of the environmental air to intrude into the TC inner-core was through the frictional inflow layer, and not through the mid-levels. These studies act as building blocks for the present study and also serve as cautionary tales that the conclusions that we draw are manifestations of the approaches and frameworks we use. Real TCs warrant further understanding where the asymmetric structure of winds in three dimensions is much more complex than those prescribed in the above studies.

The Bay of Bengal basin differs from its Atlantic or Pacific counterparts in that the ocean is warmer^36,37^, shallower, and smaller in size. As a result, the atmospheric environment plays a dominant role in dictating TC behavior^38^. The primary objective of this manuscript is to bring attention to the vastly different environments experienced by TCs over the Bay of Bengal within a relatively short period of time and how such differences are strongly tied to the differences in the convective organization of the two TCs under consideration. The differences in the environments experienced by Phailin and Lehar nicely set up the stage for the analysis of the drivers of the evolution of the convective organization in a low-sheared storm versus a sheared storm. Since convection is by and large a stochastic process and occurs at a multitude of scales^39^, the central question of interest here is: How do the large-scale TC environments set-up the local environments within the vortex such that they are supportive or disruptive to the *growth and sustenance* of deep convection?

## Results

**Figure 1:**
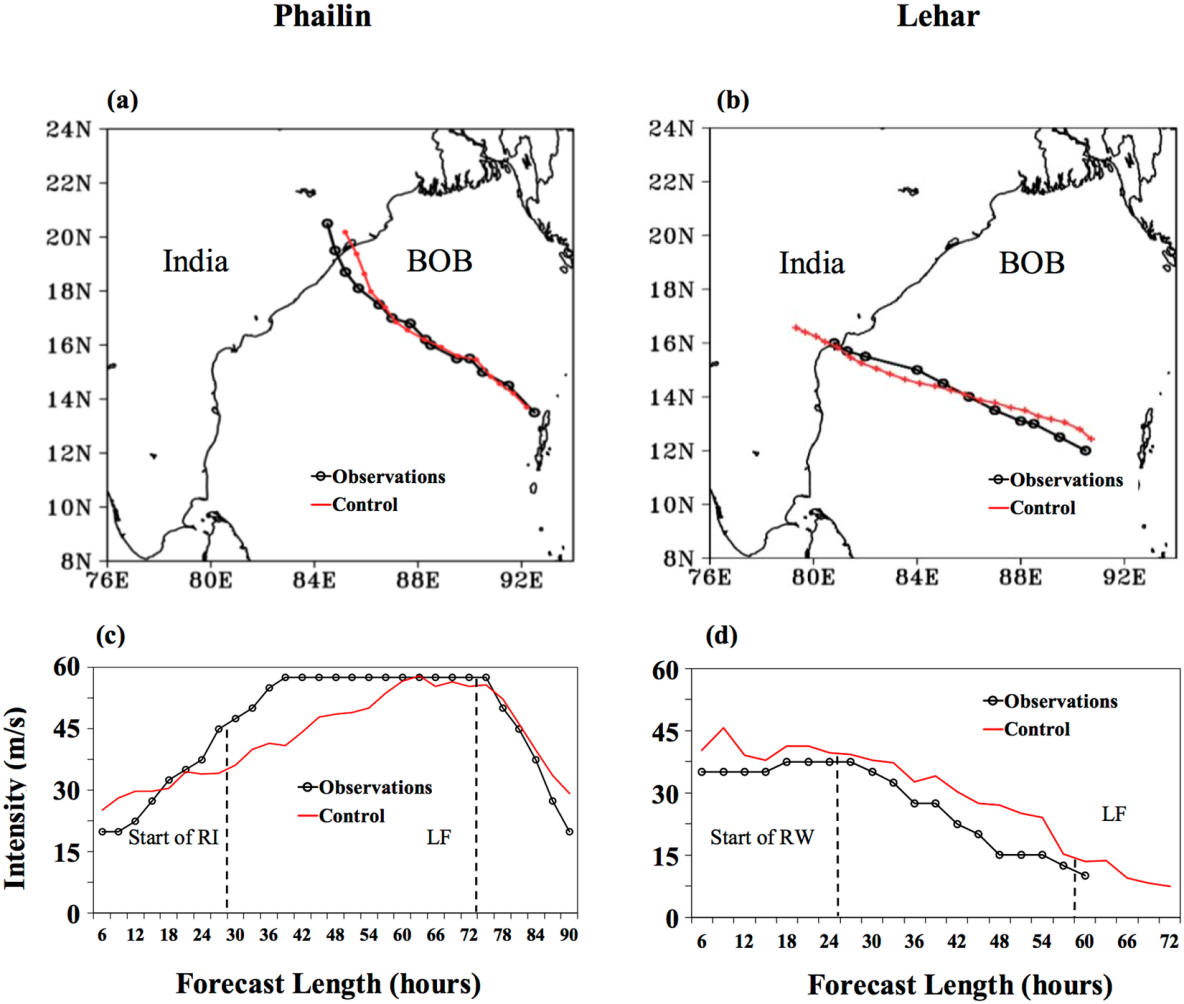
Comparison of model-simulated tracks (**a**) Phailin initialized at 12 UTC, 09 October 2013 and (**b**) Lehar initialized at 00 UTC, 26 November 2013. **(c**,**d**) Are the same as (**a**,**b**) but for intensity where the start of RI/ RW, and the landfall points are indicated with dashed lines. BOB: Bay of Bengal. 1 m/s = 1.94384 knots.

Figure 1 shows the comparison of model-simulated track and intensity for Phailin and Lehar with observations from the India Meteorological Department (IMD). The differences in the evolution of the tracks (Fig. 1a,b) and life spans of these TCs were small compared to the differences in their intensity evolutions. Phailin rapidly intensified by about 36 m/s (from 23 to 60 m/s; ∼70 knots) between 10 and 11 October 2013 and Lehar decayed by about 15 m/s (from 40 to 25 m/s; ∼30 knots) between 27 and 28 November 2013 before weakening a further 9 m/s (∼17.5 knots) over the next 12 hours (Fig. 1c,d). Both these rapid intensity changes began when the TCs were nearly at the same proximity to land. We adopt the following methodology. We first compare the evolution of the environments that the TCs are subject to, then compare the evolutions of the dynamic-thermodynamic variables within the TC vortex, and finally the channels through which external and internal physical processes interact.

### Comparison of TC environments

**Figure 2:**
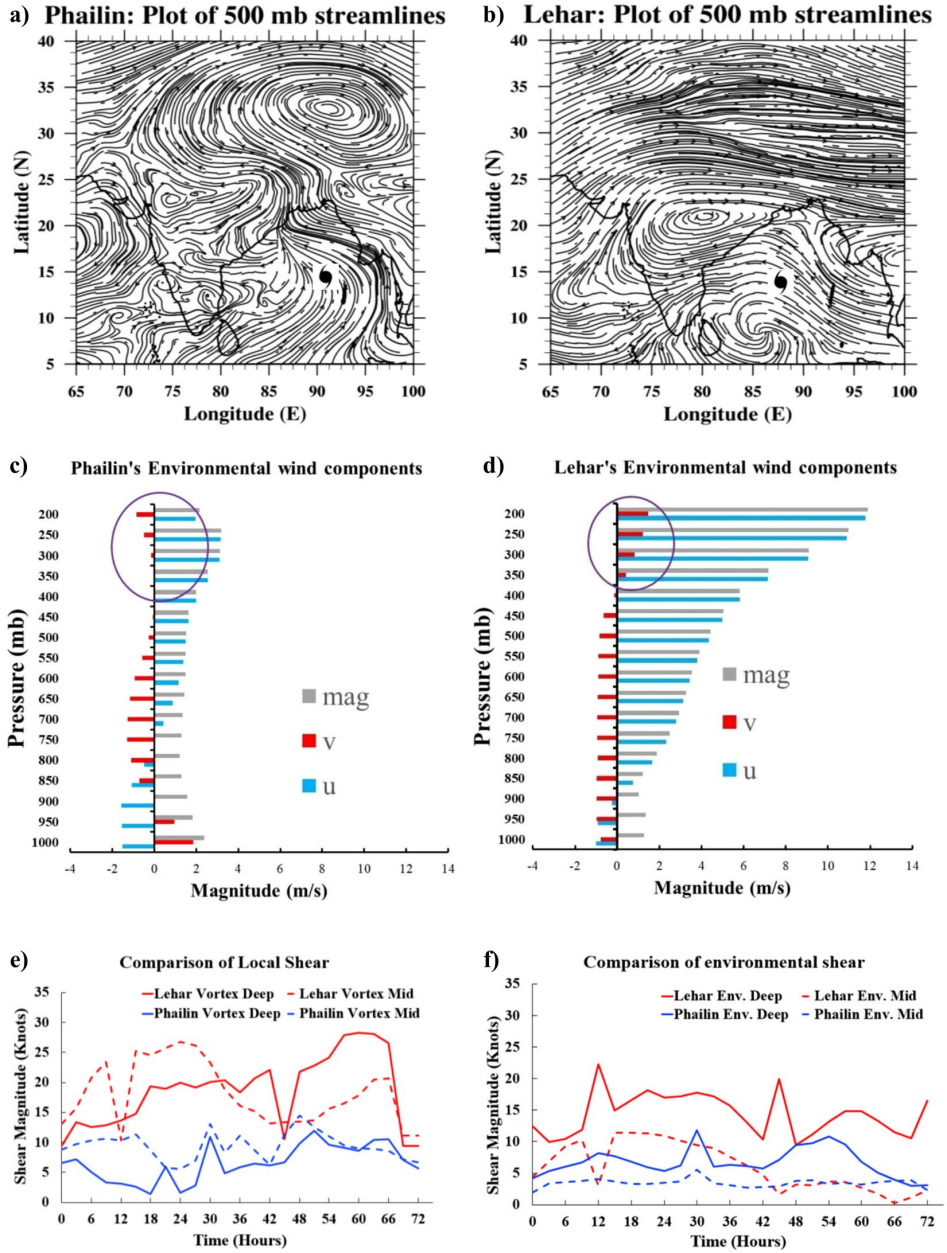
(**a**,**b**) Are plots of (vortex-removed) streamlines at 500 mb for Phailin and Lehar, showcasing the position of the subtropical high. (**c**,**d**) Present a snapshot (t = 24 hours) of the environmental zonal wind, meridional wind, and the wind vector magnitude, computed at each level for Phailin and Lehar (averaged within an 8.5° × 8.5° box after vortex removal). The highlighted portion indicates the presence of an upper-level anticyclone (positive and clockwise tangential velocity) in Lehar’s environment and the absence of it in the case of Phailin. (**e**,**f**) Serve to compare the deep (200–850 mb) and mid (500–850 mb) bulk-shear values within the vortex (the winds at each level are first domain averaged in an 2° × 2° box) and in the environment (the vortex is removed and the domain averaging of winds at each level is done within an 8.5° × 8.5° box).

Figure 2 shows the differences in the large-scale environments of Phailin and Lehar at the times where the storms began to diverge in their intensities. The plot of 500 mb streamlines (vortex-removed) at t = 27 hours(start of RI) for Phailin (Fig. 2a), reveals a large subtropical anticyclonic ridge over the Tibetian-Himalayan region (Lat 27–40°). The mean position of the subtropical high shifts southward each month between July and February (Supplementary Figure 2) and its trajectory is in synchronization with the propagation of the monsoonal heat low (cf. Figure 5.4 Krishnamurti *et al*.^40^). In the case of Phailin, the subtropical high was anomalously north, and comparable to what is expected in September, as per climatology (Supplementary Figure 2). This anomalous position of the subtropical high is potentially linked to the variability in the withdrawal of the monsoons in the year 2013. An active period in late September-October 2013, delayed the withdrawal by ∼3 weeks, with the withdrawal isochrones differing by ∼10–15° during the time that Phailin (the first “post” -monsoon TC of 2013) traversed across the Bay of Bengal^41^.

On the other hand, the dominant anticyclonic ridge had moved southward and away from the Himalayas (∼Lat 17–22°) by mid-November (Fig. 2b). This is consistent with the post-monsoon, early-winter climatology (cf. Supplementary Figure 2). Apart from the seasonal progression of the subtropical ridge within six-weeks, Lehar’s environment was potentially influenced by its predecessors Phailin and Helen (not explored in this study). Figure 2b indicates that the north-south pressure gradients on either side of the ridge were large, causing the ridge to be compressed around its center. Figure 2c,d compare the environmental wind components (vortex removed, domain averaged in an 8.5 × 8.5° box around the center) for each of the TCs. Figure 2c,d show the depth and intensity of the environmental winds impacting the TC vortex. Figure 2d indicates the presence of an upper-level anticyclone, extending to about 400 mb, six hours before the start of rapid weakening. While the figure only shows a snapshot in time, analysis of these streamlines with depth across several times, indicated a tilted ridge, extending down to 650 mb at certain times. Further, the ridge presented as two bifurcated centers at the start of Lehar’s lifecycle and then went to evolve into a giant anticyclone by the end of its life-cycle (not-shown). The proximity (∼10° from the TC), intensity, and depth of the upper to mid-level anticyclone cause the upper portions of the vortex to experience a different environmental forcing as compared to the lower portions of the vortex. This manifests as shear in Lehar’s environment. Overall, the intensity and magnitude of the winds impacting Phailin (Fig. 2c) are much less than that of Lehar.

Figure 2e,f show the comparison of the evolution of local- (bulk) shear (computed using the vector difference of domain averaged winds within a 2° × 2° box, without removing the vortex) and environmental (bulk) shear (computed using the vector difference of domain averaged winds within a 8.5° × 8.5° box, after the vortex is removed; Further details in Methods section) for Phailin and Lehar. The motivation behind differentiating behind the environmental and local-shear is two-fold. First, the differentiation allows us to move away from the traditional consideration of shear as a one-way forcing from the environment onto the vortex, and acknowledge that the vortex is feeding back onto the environment and (potentially) modifying its own local-shear. Second, there are multiple definitions of shear in use within the TC community while conducting shear-vortex experiments (e.g., the 120 km domain used in Riemer *et al*.^31^ v/s the vortex-removed, 500 km domain in Chen and Gopalakrishnan^26^). These differences are due to an implicit understanding within the community that the shear “experienced” by the vortex, is possibly different from the environmental-shear (Personal communication with Riemer, M., Ryglicki, D., and Rogers, R).

Figure 2e shows that the deep-shear of Lehar was persistently between 15–20 knots before and during the period of rapid weakening (16–40 hours) and ≥20 knots as Lehar approaches land, and post-landfall (48–72 hours). Furthermore, Fig. 2e reveals that the mid-shear was particularly high (∼25 knots) between 12–30 hours when the rapid weakening was initiated. On the other hand, Phailin’s local-shear was consistently below 10 knots, except for a modest increase towards the end (45–66 hours) when the storm was at peak intensity. Figure 2e,f also indicate clear differences between the environmental-shear and local-shear, despite structural similarities. For example, the magnitudes of mid-shear within the vortex are nearly double the magnitudes in the environment between 12–42 hours, despite similarities in their trends. The magnitudes of Lehar’s environmental-shear are comparable to that of its local-shear magnitudes between 12–42 hours when the storm rapidly weakened but are much lower between 48–72 hours. While Phailin’s environmental deep-shear values are slightly higher than its local counterpart, its environmental mid-shear values are almost half of its local-shear values.

**Figure 3:**
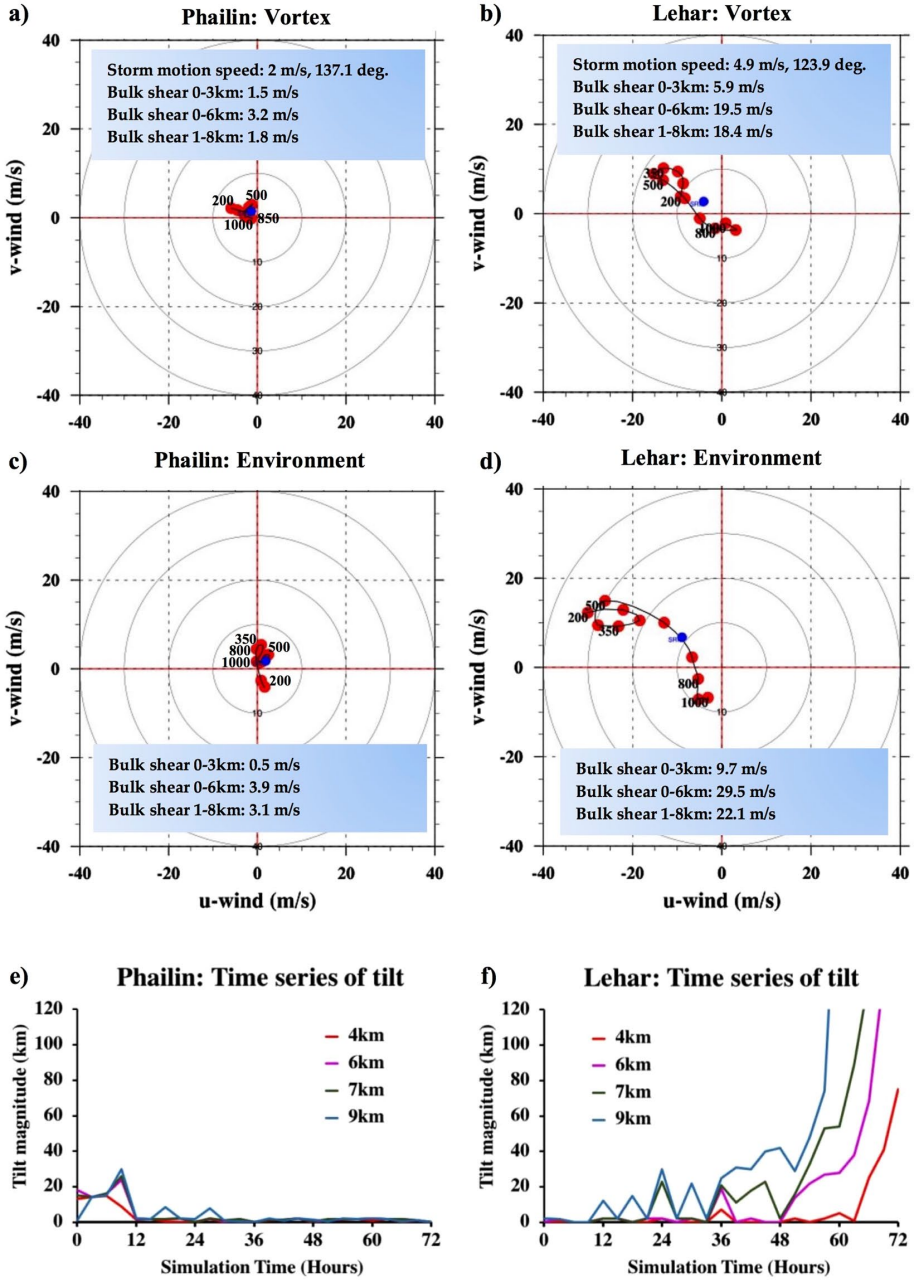
(**a–d**) Hodographs of winds computed within the vortex (domain averaged in an 2° × 2° box at each vertical level) and in the environment (domain averaged in a 8° × 8° box after vortex removal at each vertical level) for Phailin just before the RI (t = 27 hours) and for Lehar just before the RW (t = 27 hours). The shear magnitudes are vector differences between domain averaged winds computed at the vertical levels indicated. (**e**,**f**) Time series of vortex tilt (difference in circulation centers at various levels from the circulation center at 1.5 km) for Phailin and Lehar.

The traditionally used bulk-shear estimates (the difference between wind vectors at 200 mb and 850 mb for deep-shear, and 500 mb and 850 mb for mid-shear) might under-represent the structural complexity of the vortex and the environmental flow-field in three dimensions. For this purpose, we present the hodographs of winds computed within the TC vortex and in an annulus outside the vortex, representative of the environment (Fig. 3a–d) right before the start of the rapid change in intensity, for each of the TCs. Also highlighted are the storm motion speed and direction, and the bulk-shear computed at different levels. Figure 3a indicates an organized vortex, with very low shear within the vortex and the environment (cf. Fig. 2e,f at t = 24 hours). Compared to Phailin, Lehar’s wind magnitudes are more than twice as greater within the vortex, and close to four times greater in the far environment, and the flow-field is far more complex (Fig. 3b,d). Figure 3b serves to illustrate that the bulk-shear estimate using the difference between 200 and 850 mb might be under-representing the actual shear in the vortex. For example, at t = 24 hours (just before the start of the weakening), the maximum shear is between 350 mb (∼8 km) and 850 mb at 18.4 m/s or 35.76 knots. Figure 3d is indicative of the intense (≥20 m/s or 38.8 knots), southeasterly winds between 200 and 500 mb due to the proximate ridge. Figure 3e,f are plots of the time-series of the vortex tilt (see Methods section for details) between the circulation centers at 1.5 km with various heights. Phailin’s time-series shows that after an initial fluctuation in the tilt (with magnitudes extending to ∼30 km), the amplitude drops considerably at around 12 hours after which, the tilt is negligible. On the other hand, Lehar’s plot of tilt indicates an increasing discordance between the circulation centers at different heights (especially after ∼36 hours). As suggested in Reasor *et al*. (2004), there is a clear oscillation of the TC vortex in pursuit of dynamic equilibrium with its environmental forcing. While the frequency of the oscillation seems steady at around 6 hours, the amplitude of the oscillation increases as the ability of Lehar’s vortex to realign vertically reduces with time. As the tilt extends to various depths of the vortex, like a feebly spinning top conserving angular momentum, the amplitude of tilt gradually increases as the vortex weakens.

### Comparison of vortex-scale thermodynamics

**Figure 4:**
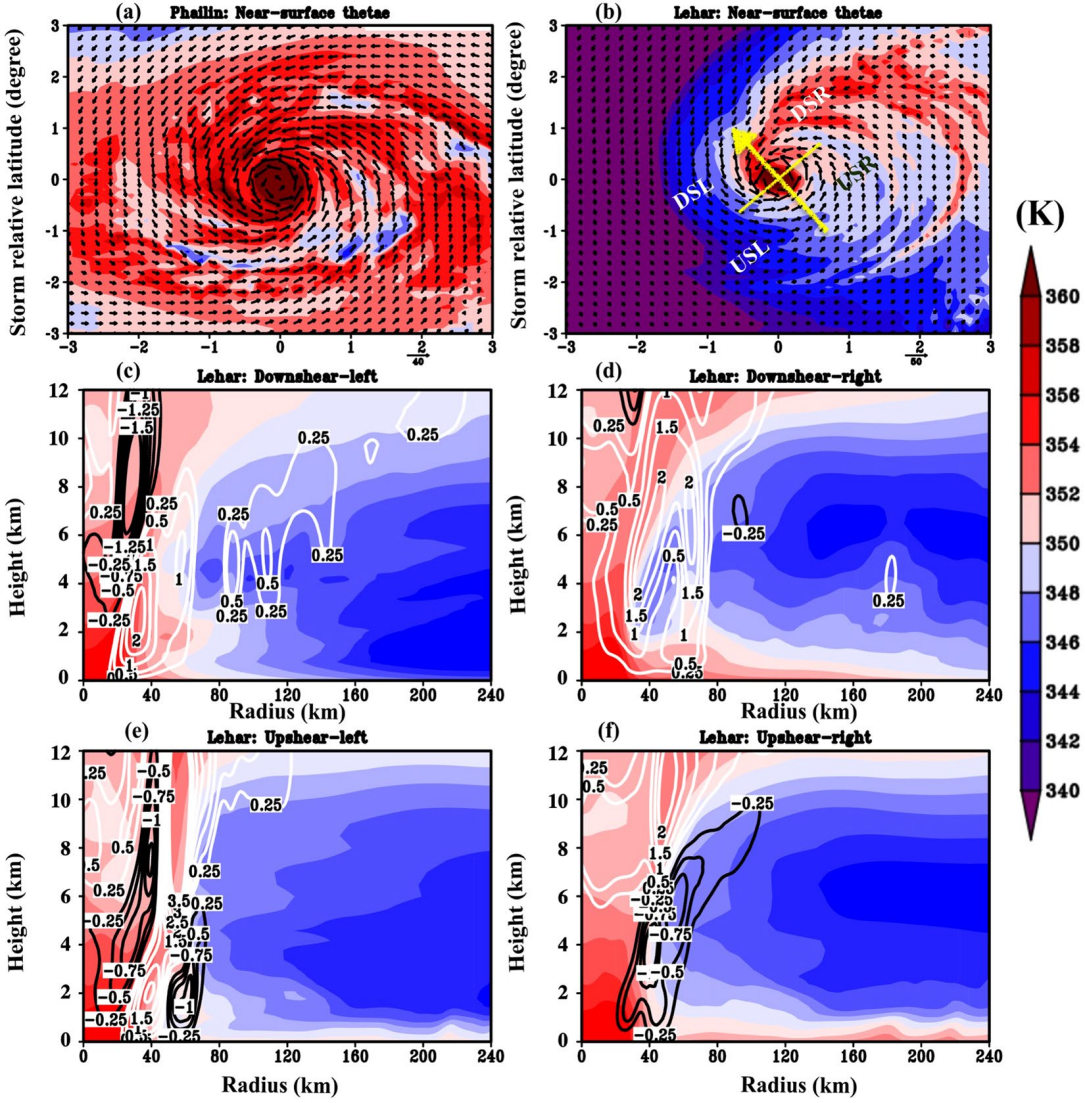
Plan view of the near-surface equivalent potential temperature for Phailin (**a**) and Lehar (**b**) three hours prior to the start of the rapid intensity change. The four quadrants - downshear right (DSR), downshear left (DSL), upshear left (USL) and upshear right (USR) are marked in (**b**). (**c**–**f**) Present the quadrant averaged, radial-height plots of *θ*_*e*_ for Lehar at t = 24 hours. The white contours indicate upward motion and the black contours indicate downward motion.

Following the analysis of the large-scale environmental flow field, in the following section, we present an analysis from the perspective of the vortex such that the key takeaways are applicable in multiple scenarios beyond the case studies presented here. Our emphasis will be to contrast the aggregate impact of the organization of convection in Lehar’s sheared vortex with that of Phailin’s low-sheared vortex. We seek to understand the configuration necessary for persistent, deep convection and how this is attained in Phailin and disrupted in Lehar. To obtain a picture of the (a)symmetric convective processes, a plan view of the near-surface boundary layer *θ*_*e*_ (averaged within 2 km from the surface) is shown for Phailin and Lehar in Fig. 4a,b respectively. Figure 4a reveals an envelope of high *θ*_*e*_ around Phailin’s eyewall. On the other hand, Fig. 4b underscores an azimuthally asymmetric distribution of Lehar’s *θ*_*e*_, with the highest near-surface *θ*_*e*_ concentrated in the downshear right quadrant (DSR), consistent with prior observational^11,42^ as well as modeling studies^31^.

#### Vortex-scale thermodynamics in a sheared, weakening storm

To further understand the azimuthal and radial distribution of updrafts and downdrafts in Lehar’s sheared vortex, the radius-height plot of each of the quadrants highlighted in Fig. 4b is shown at the start of the weakening process (Figures c–f). The deep convection (we define this as the upward motion in which the convective mass flux is transported to at least 8 km in the vertical) is concentrated mostly in the downshear quadrants - downshear left (DSL) and DSR around 40–60 km radius (Fig. 4c,d). In the upshear-left quadrant (USL), the upward motion is located ∼40 km radius between regions of strong downward motion on either side. Consistent with prior documentation^26^, strong, convective-scale downdrafts that are maximized around 8–10 km in the vertical are present in the DSL and USL quadrants, albeit within the eyewall region (radii ≤ 40 km). However, these downward motions within the eyewall region are unlikely to play any role in bringing the environmental low *θ*_*e*_ air into the storm vortex, since the eyewall is thermodynamically protected by deep convection and dynamically by the strong tangential winds at the radius of maximum winds (RMW) (30–40 km). As articulated in Riemer and Montgomery^32^, a combination of downward motion and cross-vortex (storm-relative, radial) flow is needed for the environmental air to intrude into the vortex core.

Just outside the eyewall region between 40–80 km radii, deep downdrafts in the upshear quadrants (upshear left (USL) and upshear right (USR) in Fig. 4e,f) are seen reaching the surface. These downdrafts are present underneath moderate to strong updrafts (vertical velocities between 0.5 to 1.5 m/s) and are in the proximity of low *θ*_*e*_ air. When the precipitation from these updrafts falls through these dry (low *θ*_*e*_) unsaturated regions, it is expected to evaporate. The downdrafts (sinking motion) seen in the upshear quadrants are most likely a result of the consequent cooling that occurs when the latent heat of evaporation is subtracted from the surrounding environment as addressed in several previous studies^25,31,33,34,43,44^. Riemer *et al*.^31,45^ noted in their idealized experiments that such downdrafts might act to bring the low *θ*_*e*_ from outer radii (e.g. radii greater than 80 km in this case) into the boundary layer and near-surface regions.

**Figure 5:**
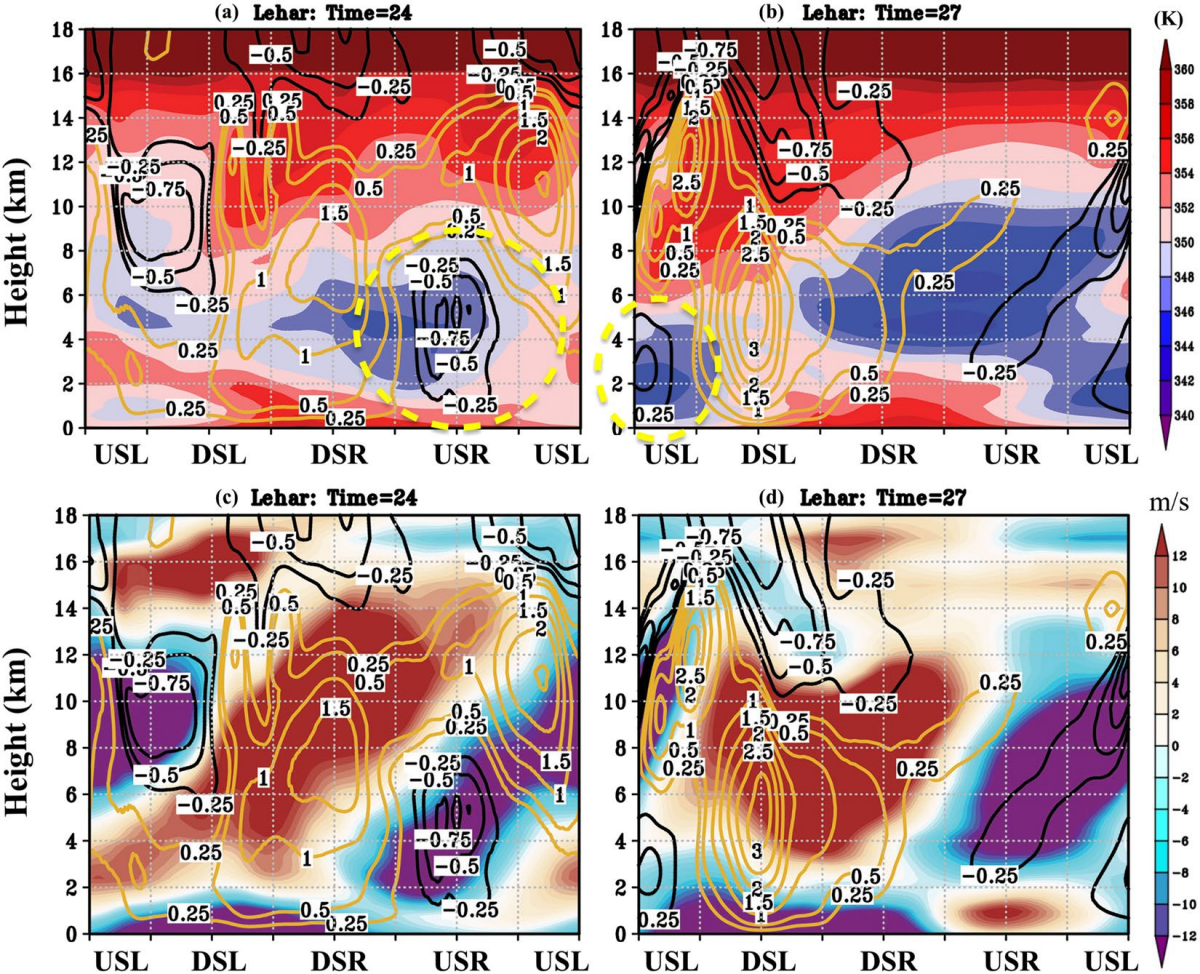
Lehar’s Azimuth-height plots of *θ*_*e*_ (shaded) and vertical velocity contours (updrafts in golden and downdrafts in black) at two times: t = 24 hours (3 hours before RW) and t = 27 hours(start of RW) (panels a,b respectively). Figure 5c,d show the corresponding azimuth-height curtains for radial velocity (shaded; negative values represent inflow and positive values represent outflow) and vertical velocities (contours) for Lehar at the same times.

However, both the upshear quadrants contain such downdrafts. This begs the question: Which of the two upshear quadrants present a likely configuration for the intrusion of low moist entropy air? The answer to this depends on the relative location of the downdrafts carrying low *θ*_*e*_ air (through the top of the boundary layer) with respect to the location of the inflow (within the boundary layer). We present an azimuth-height plot in Fig. 5 while radially averaging between 20–80 km, keeping in mind that the radial location of these downdrafts was between 40–80 km (Fig. 4e,f) and that the RMW (at the surface) was at 30 km. Figure 5a,b show Lehar’s *θ*_*e*_ (shaded) and vertical velocities (contours) at two times: t = 24 hours, that is three hours before the weakening and at t = 27 hours, when the rapid weakening began. Likewise, Fig. 5c,d show the radial velocity (shaded) and vertical velocities (contours) for the same times as Fig. 5a,b. The *θ*_*e*_, the vertical velocities, and the radial velocities are all very asymmetric in the azimuthal direction, consistent with the behavior of sheared storms in Marks *et al*.^46^, Rogers *et al*.^11^, Reimer *et al*.^45^ and others. Additionally, in Lehar’s case, the direction of storm motion was coincident with the direction of deep-shear. While we know that a combination of these vectors dictates the azimuthal distribution of inflow^20,21^, the asymmetries due to motion and those due to shear may interfere destructively or constructively, and a comprehensive understanding of their nonlinear interplay is yet to be reached (a topic not explored in this study). As expounded in the previous sections, the vertical structure of the environmental winds might be much more complex than that indicated by the bulk-shear vectors. From that perspective, while we have a general understanding that downdrafts (updrafts) occur in the upshear (downshear) side and that inflow (outflow) occurs in the upstream/downshear (downstream/upshear) side^14,46^, our prior knowledge based on the bulk (deep) shear vector might fail to capture the complexity in the spatial distribution of the downdrafts, inflow, and *θ*_*e*_ for specific cases.

In Fig. 5a,b, the downdrafts carrying low *θ*_*e*_ air are maximized in the USR (NE-E-SE) quadrant at t = 24 hours but move to USL (SE-S-SW) by t = 27 hours. These azimuth-height ‘curtain’ plots may be understood along the direction of the flow, i.e., cyclonic (DSR-DSL-USL-USR-DSR or NE-NW-SW-SE in this case; cf. Fig. 5b). Given that these plots are snapshots in time, they must be cautiously interpreted as a spectrum of updrafts and downdrafts that combine to produce a net effect indicative of updrafts or downdrafts. Single updrafts/downdrafts are not tracked here, and it is possible that individual downdrafts exist in the presence of mean upward motion and vice versa. At t = 27 hours, the updrafts can be seen to begin in the DSR quadrant (NW-NE-E) and maximize in the DSL quadrant (SW-NW). Further, upper-level updrafts overlay a low-level downdraft in the USL region (S-SW, between 6–16 km in Fig. 5b). This is very consistent with prior observational studies^11,16,18,47^ where an updraft was seen to initiate DSR, mature DSL, ascend USL, whilst overlaying low-level downdrafts, before terminating USR. The convective patterns in t = 24 hours is a little more complicated, possibly because we are taking a snapshot when the convection is at different stages. At this time, there is a strong updraft in the DSR (W-NW-N-NE) quadrant, a weak mid-to-upper-level downdraft DSL/USL (W-SW-S-SE), a weak updraft USL (SW-S) and a strong downdraft USR as mentioned above. Between the two times, the inflow structure remains almost the same (Fig. 5c,d). A low-level inflow is prevalent in the S-SW-W-NW or USL-DSL-DSR region, and an upper-level slanted outflow is present in the W-NW-N-NE or DSL-DSR regions. Further, a more azimuthally confined region of low-level outflow is prevalent in the NE-SE (USR) quadrant. While there is a slight extension of the low-level outflow in the SE-S (USL) quadrants at t = 24 hours, the low-level outflow is confined to the USR quadrant (NE-SE) at t = 27 hours, and low-level inflow occupies the USL quadrant (SE-S-SW).

**Figure 6:**
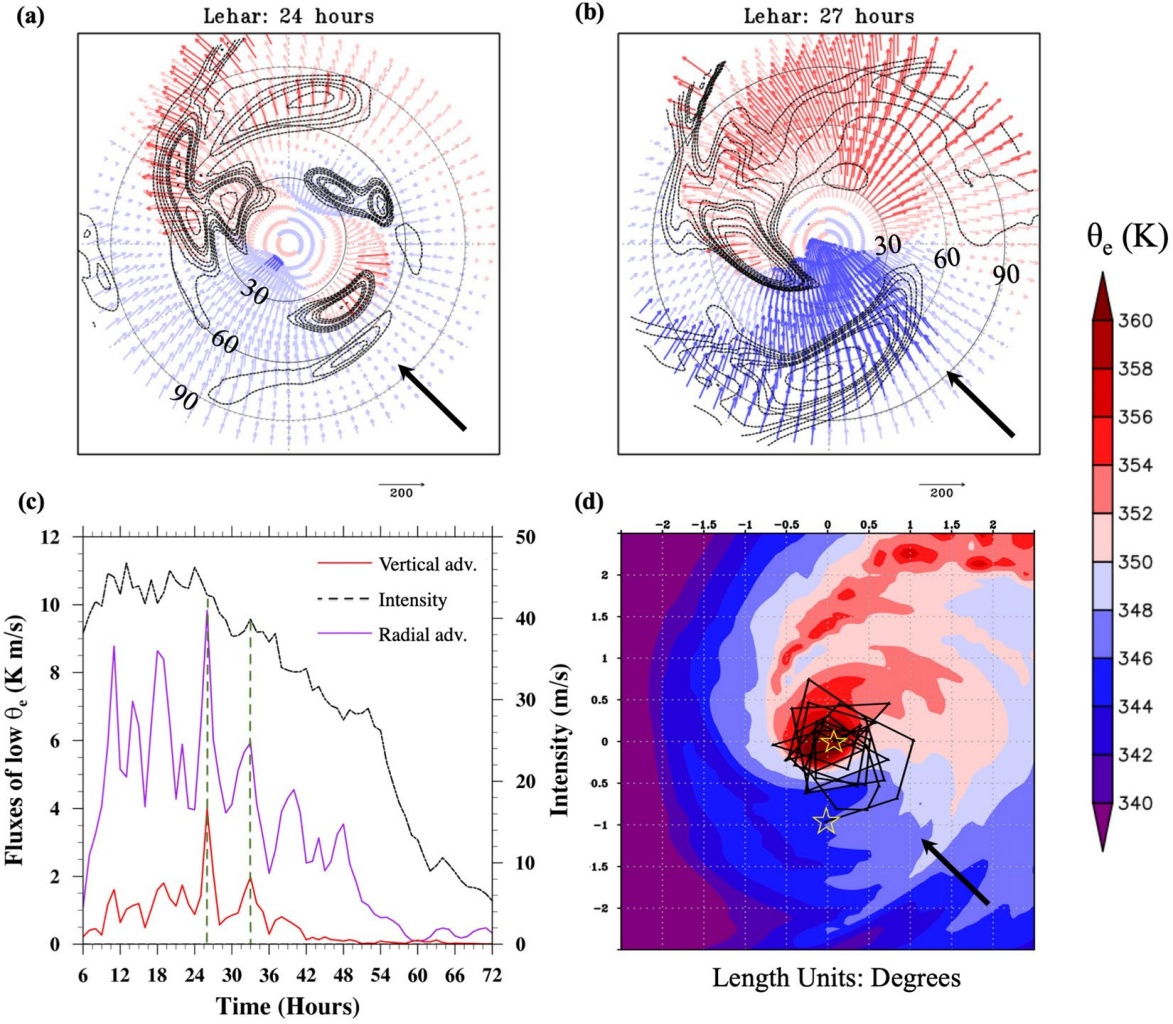
(**a**,**b**) Radial flux of *θ*_*e*_ anomaly $(u{\theta ^{\prime} }_{e})$ within the boundary layer (vectors, colored according to ${\theta ^{\prime} }_{e}$ (azimuthal mean is subtracted) where blue indicates negative ${\theta ^{\prime} }_{e}$ and red indicates positive ${\theta ^{\prime} }_{e}$). The ${\theta }_{e}$′ is averaged from the surface to 1.5 km in the vertical to represent the *θ*_*e*_ distribution within the boundary layer. Additionally, the downward flux of ${\theta ^{\prime} }_{e}$ $(\,-\,{1}^{\ast }w{\theta ^{\prime} }_{e})$ through the top of the boundary layer (assumed to be at 1.5 km) is indicated as dashed contours. These plots are shown at two times: t = 24 hours (just before RW) and t = 27 hours(start of RW). (**c**) Time-series of the magnitudes of $u{\theta ^{\prime} }_{e}$ and $w{\theta ^{\prime} }_{e}$ where a Heaviside function is used to ensure that only the inflow, downdrafts, and negative ${\theta ^{\prime} }_{e}$ are considered in the computation. The times when the magnitudes of the vertical and radial fluxes of low *θ*_*e*_ peak together are highlighted and shown in the context of the timing of the rapid weakening. (**d**) Forward-trajectories of an air parcel with low-${\theta ^{\prime} }_{e}$ (blue region) initiated at a random point in the upshear left quadrant at t = 27 (marked by a star at 80 km radius). The end point of the air-parcel is indicated by a star close to the TC center. The radial extents for panels a and b are 0 to 90 km and are indicated in the figures. The deep-shear vector is indicated by the black arrow.

The distribution of *θ*_*e*_ within the boundary layer is dictated by the vertically downward (mean and eddy) fluxes through the top of the boundary layer, the radially inward (mean and eddy) fluxes within the boundary layer and the surface fluxes^34,42,44^. Our focus in this section (Figs 5 and 6) is specifically on the downward and radially inward eddy (azimuthal mean field is subtracted) fluxes of low *θ*_*e*_. At t = 24 hours, when the downdrafts are maximized in the USR quadrant (Fig. 5a), the vertical flux of low *θ*_*e*_ air through the top of the boundary layer is juxtaposed predominantly with the radially outward flux of low *θ*_*e*_ within the boundary layer (Figs 5c and 6a). Under such a scenario, one would expect that the low *θ*_*e*_ air flushed into the boundary layer by the downdrafts simply exits the region without penetrating into the eyewall. On the other hand, at t = 27 hours when the downdrafts are in the USL quadrant (Fig. 5b), they are collocated with inflow within the boundary layer (Fig. 5d). In other words, the vertical eddy flux of low *θ*_*e*_ air through the top of the boundary layer is juxtaposed with radially inward eddy flux of low *θ*_*e*_ air within the boundary layer (Fig. 6b). Such a juxtaposition in the upshear left quadrant creates a configuration that is conducive for the low *θ*_*e*_ air from the environment to penetrate into the boundary layer and then into eyewall region (as evidenced by the blue, radially inward vectors reaching inner radii). Figure 6c provides a time-series of the (azimuthally and radially averaged) magnitude of vertical and horizontal fluxes transporting low *θ*_*e*_ air. Figure 6c illustrates that in addition to the collocation in the azimuthal phasing evidenced in Figs 5a–d and 6a,b, there was synchronization between the peaks of *magnitudes* of the vertical and radial fluxes of low *θ*_*e*_ air. Finally, Fig. 6d shows the forward trajectories of an air parcel whose *θ*_*e*_ value was below 342 K initiated randomly in the USL quadrant at around 80 km radius where the downdrafts were predominant. The trajectories of the low *θ*_*e*_ air are seen to intrude into the inner-core of the TC vortex.

#### Vortex-scale thermodynamics in a low-sheared, intensifying storm

The diagnostic developed for Lehar where the azimuthal variations in its dynamic-thermodynamic fields were large, may not be applicable for Phailin, where the azimuthal variations in the same fields are weak in the absence of shear. The following section seeks to explore the alternative diagnostics applicable for TCs such as Phailin where azimuthally *symmetric* convective organization is prevalent within the vortex.

**Figure 7:**
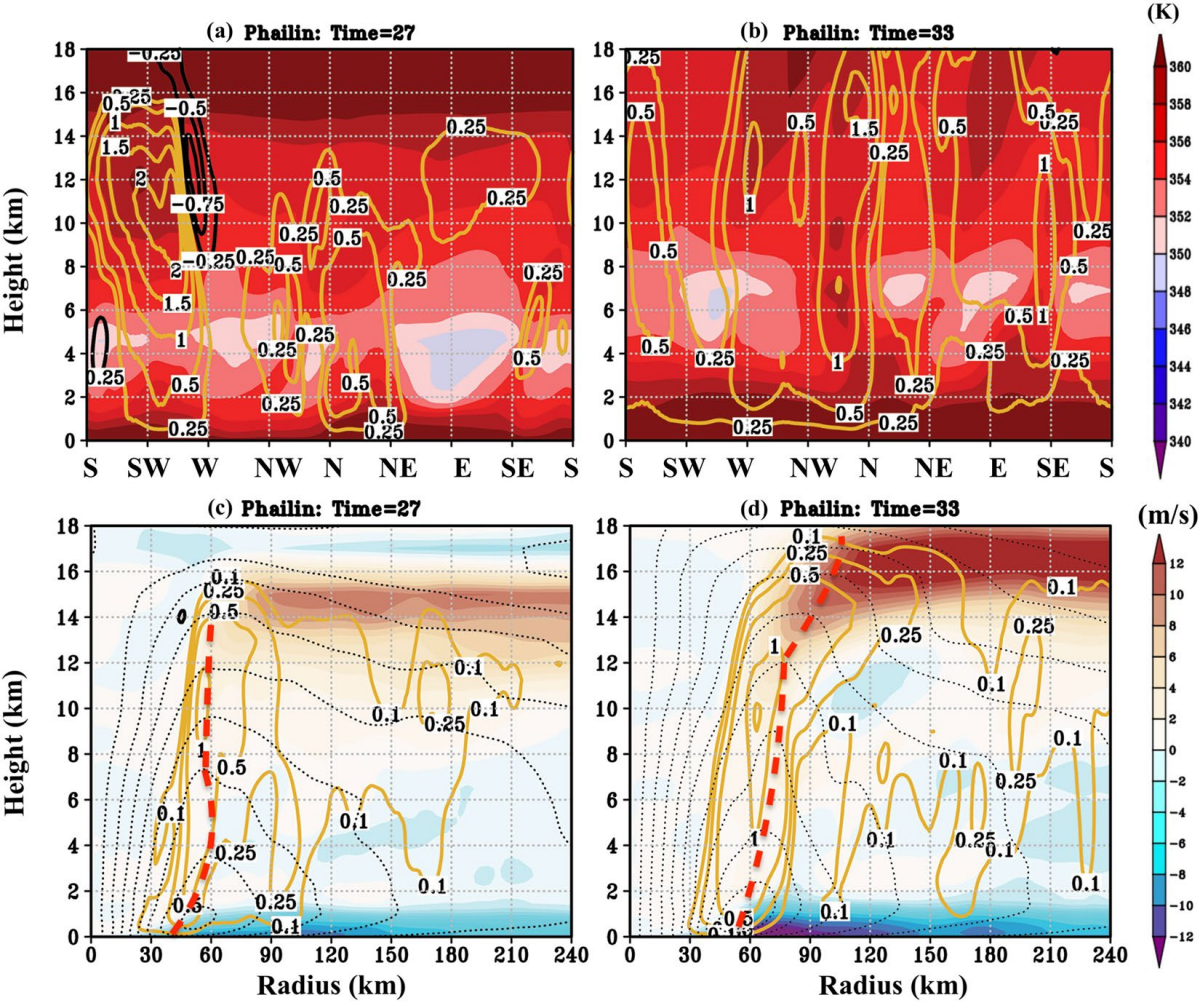
(**a**,**b**) Azimuth-height plots of Phailin’s *θ*_*e*_ (shaded) and vertical velocities at t = 27 hours (six hours before the RI) and at t = 33 hours (start of RI). (**c**,**d**) Present the azimuthal-averaged, radial-height cross-section of Phailin’s radial velocities (shaded; negative values represent inflow, and positive values represent outflow)for the same times. Also highlighted are the RMW (red, dashed lines) at each height.

Figure 7a,b show the plot of *θ*_*e*_ (shaded) and vertical velocity contours (radially averaged on either side of the RMW) six hours before RI (t = 27 hours) and at t = 33 hours (start of RI). What is consistent between the two times, is the presence of relatively high *θ*_*e*_ when compared to Lehar (Fig. 6a,b). At t = 27 hours, there are strong bursts of deep convection extending up to 16 km in the west to south (W-S) quadrant and 14 km in the northeast to northwest (NE-NW) quadrant. Six hours later, this deep convection has wrapped around and extended to all the quadrants - a feature that is entirely absent in Lehar. In Lehar, the initiated updrafts die down in the upshear quadrants where the thermodynamic environment is unfavorable (due to reduced *θ*_*e*_ and the predominance of evaporation-induced and/or tilt-driven downdrafts). This signature of deep convection wrapping around in rapidly intensifying TCs has been noted previously in several studies^19,26,47–50^. Unlike Lehar, the azimuth-height perspective of radial velocity does not offer much insight for symmetric TCs such as Phailin. This suggests that symmetry dominates in Phailin as opposed to Lehar being dominated by asymmetry.

Figure 7c,d present the azimuthal-averaged, radial-height cross-section of Phailin’s radial velocity at times, 27 and 33 hours. They serve to illustrate the importance of the inflow magnitude and radial profile of convergence within the boundary layer. Also highlighted, is the RMW for each of the times. The time-series of near-surface RMW for Phailin (Supplementary Figure 5a) indicates that Phailin underwent an eyewall replacement cycle with the RMW fluctuating from 42 at t = 27 hours to 35 at t = 30 hours, before rising to 56 at t = 33 hours (See Supplementary Figure 5b for variation of RMW across different heights between these times). While one might expect that the RMW shrinks during intensification in a prototypical intensification problem (following angular momentum conservation arguments), an eyewall replacement cycle can cause the RMW to expand and the TC to intensify at the same time. According to Shapiro and Willoughby^51^, either a heat source (such as convection) or a momentum source (such as inflow within the boundary layer) can cause the RMW to contract or expand. For example, if there is an additional heat source (convection) at an outer radius during a secondary eyewall formation, it is entirely plausible that the RMW expands and the tangential winds increase at the same time. Further, it must be noted that the angular momentum within the boundary layer is not conserved. Therefore, a more reliable method of understanding the intensification process is using the evolution of azimuthally-averaged, angular momentum surfaces (M-surfaces, see Figure 13 in Montgomery and Smith, 2014)^52^. Supplementary Figure 6 serves to demonstrate that the angular momentum surfaces move inwards in the case of Phailin, as the TC intensifies regardless of the expansion in its RMW. Note that this expansion in the RMW during the RI period is consistent with the conclusions of Stern *et al*.^53^ although their focus was mainly on the ceasing of the contraction of the RMW rather than its expansion.

A key point to note is that while Phailin’s absolute radius of the location of deep convection did not vary much between t = 27 hours and t = 33 hours, the relative location of the convection with respect to the RMW was far more radially inward at t = 33 hours (Fig. 7d), as compared to t = 27 hours (Fig. 7c). On the other hand, the radial location of the peak inflow within the boundary layer, shifted outward when the magnitude of inflow increased by about 6 m/s (Fig. 7c,d). A possible (but neither definitive nor exhaustive) explanation of why the RMW might expand during the RI period might be that with increasing mass flux, the area required to support such a magnitude of mass flux must increase. Ergo, the RMW expands to support such an intensification beyond a certain stage.

**Figure 8:**
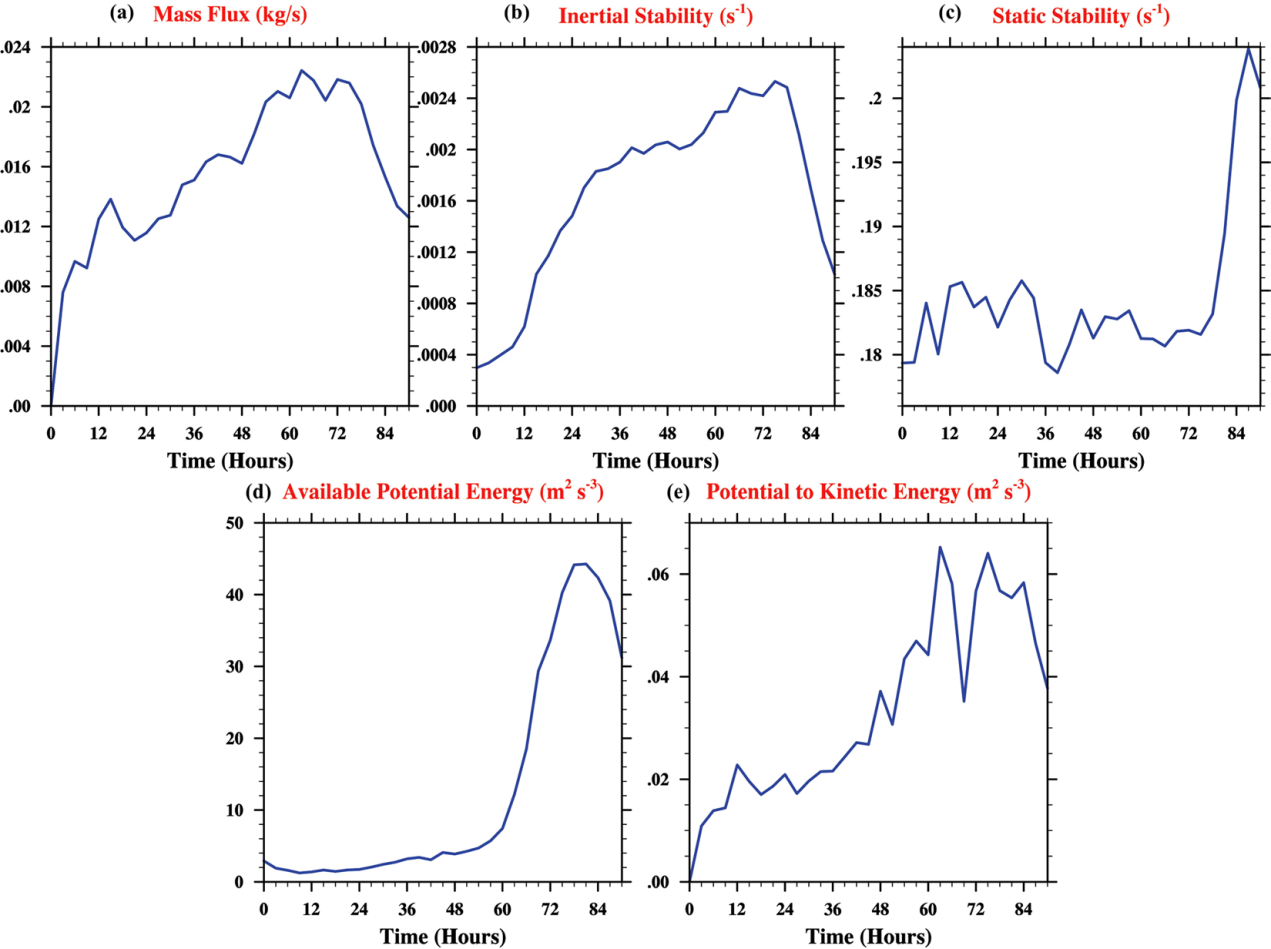
Time-series plots (averaged in the azimuthal, radial and vertical directions) for Phailin within the RMW. (**a**) Vertical Mass flux in kg/s (**b**) Inertial stability, *I*^2^ (*s*^−1^) (**c**) Static stability, *N*^2^ (**d**) Generation of available potential energy (*m*^2^*s*^−3^) (**e**) Conversion from potential to kinetic energy (*m*^2^*s*^−3^).

Previous studies have demonstrated that the region within the RMW offers a more conducive environment for deep convection to develop and sustain, resulting in RI^54,55^. Figure 8 builds on these studies and serves to explain as to why the convection occurring within the RMW is particularly effective in spinning up the vortex rapidly. Figure 8a first shows the time-series of the magnitude of mass flux that is being advected upward within the RMW. As the convection wraps around (cf. Fig. 7b), there is increased symmetry in latent heating and more mass flux is advected upwards. This increased mass flux is indicative of stronger secondary circulation (and convection in an aggregate sense). Following hydrostatic arguments, an increased mass flux advection away from the surface suggests a drop in near-surface pressure within the inner-core. This leads to an increase in the radial pressure gradient within the boundary layer and subsequently, stronger inflow (cf. Fig. 7d). The magnitude and radial location of this inflow within the boundary layer are strongly influenced by boundary layer dynamics^6^.

Figure 8b,c show the time-series of inertial and static stability within the RMW respectively. Figure 8b,c affirm that the RMW is a region of high inertial (Fig. 8b) and low static stability. Thus, during the RI phase, when the convection occurs in this region of high inertial and low static stability, there is a higher horizontal and lower vertical resistance for the air parcels^54,55^. This results in increased upward motion that reflects as increased available potential energy (Fig. 8d) and an increased conversion from potential to kinetic energy (Fig. 8e), indicating the increase in the spin of the TC vortex (See Methods section for details).

## Discussion and Concluding Remarks

TC rapid intensity changes are the result of a combination of external and internal factors. Here, we numerically investigate the key features and forcing mechanisms in the TC environments that helped build or disrupt the organization of the convection within Phailin and Lehar. Our investigation leads to the articulation of a couple of different means to diagnose (and potentially predict) the contrasting rapid intensity changes experienced by TCs in sheared and low-sheared environments.

Our analysis of Phailin’s environment shows that the delayed withdrawal of the monsoons and anomalously northern position of the subtropical high helped establish a region of low shear. On the other hand, the environment of Lehar was baroclinic, with noticeable pressure gradients in the North-south (meridional) direction. These strong gradients compressed the flow field on either side of the subtropical ridge that had moved about 12–15° south in a span of six weeks. The increased proximity, depth, and the intensity of the anticyclone in Lehar’s environment, created a complex, sheared environment that disrupted the 3D structure of the vortex. The shear and the subsequent tilt in Lehar’s vortex are shown to be substantially more complex than that indicated by traditional bulk-shear computations. Additionally, the cold, dry continental air that was restricted to the Himalayan region in the case of Phailin was drawn south into the flow field of the ridge and Lehar.

Following the analysis of the large-scale flow field, we present an analysis from the perspective of the vortex such that the key takeaways are applicable in multiple scenarios beyond the case studies presented herein. We hypothesized that the different shear profiles had a significant impact on the symmetric or asymmetric nature of the various thermodynamic (e.g., *θ*_*e*_) and dynamic fields (e.g., vorticity) within the vortex. In Lehar’s sheared vortex, the dynamic-thermodynamic fields are shown to be asymmetric. Under such a scenario, while the demise of a vortex is the result of a series of events, we demonstrate here that the *trigger* of Lehar’s RW occurs when the azimuthal phasing of the vertical flux of low *θ*_*e*_ air through the top of the boundary layer and the radial flux of low *θ*_*e*_ within the boundary layer synchronize. Such a juxtaposition occurs in the USL quadrant between 40–80 km radii and creates a pathway for the environmental air to intrude into the eyewall region. Note that this is an extension of the boundary-layer flushing mechanism proposed by Riemer *et al*.^31,45^. We add an asymmetric component here and argue that the boundary layer flushing mechanism becomes operative only when the azimuthal phasing of the inflow is favorable to the downward flux of low moist entropy air from the downdrafts. Since convection is an intrinsic and stochastic process that occurs in response to air-sea instability, the identification of the local pockets (USL in Lehar’s case) within the vortex that are disruptive to the growth and sustenance of deep convection (in an aggregate sense) and how this ties to the evolution of the TC’s atmospheric environment is of crucial importance. Given the complexities of the shear profiles and the asymmetric distribution of the various fields within the vortex, the collocation in the azimuthal phasing and magnitude of the boundary layer inflow, vertical velocities, and low *θ*_*e*_, may be used as a diagnostic to detect rapid intensity changes. We speculate that in a sheared TC that goes on to intensify, there will be a collocation in the azimuthal phasing between the boundary layer inflow, updrafts, and positive *θ*_*e*_ anomaly. This aspect will be explored in a future study.

Conversely, in the low-sheared environment experienced by Phailin, the *θ*_*e*_, inflow, and updrafts are all distributed azimuthally in a symmetric fashion. Under such a scenario, the radial location of deep convection with respect to the RMW, the strength of the inflow, the wrapping of convection in the azimuthal direction and the transport of the momentum created in the boundary layer to the upper levels are the key elements that dictate the RI.

While the present study primarily focused on the environmental forcings and thermodynamic processes, the vortex dynamics and the role of eddy fluxes (using a tangential momentum budget) will be explored in a follow-up study. Additionally, our preliminary sensitivity experiments (not shown) indicated that the roles of the sea-surface temperature and antecedent land-surface were limited, due to the dominance of the atmospheric environment. A holistic picture warrants an analysis of the land, ocean and atmospheric forcings in addition to the vortex-scale processes. Due to the relatively small size of the Bay of Bengal basin, there is an increased likelihood of antecedent land surface variables influencing the synoptic environment, thereby influencing the intensity of landfalling TCs as they approach land. These effects will also be explored in a follow-up study with further case studies and idealized experiments, where the role of the atmosphere is not as dominant.

## Methods

TCs Phailin and Lehar were simulated using a triply-nested (27 × 9 × 3 km grid spacing), near cloud-resolving (cumulus scheme is turned off for the inner-most domain) version of the Hurricane Weather Research Forecasting model (HWRF Version 3.5a). There are 43 vertical levels, including at least 11 levels below 850 mb for adequate resolution of the hurricane boundary layer. The model is non-hydrostatically mapped on a rotated latitude-longitudinal, Arakawa E-staggered grid with a storm centered hybrid (sigma-p) coordinate in the vertical direction. This model was developed by the National Centers for Environmental Prediction (NCEP) and the Hurricane Research Division (HRD) of the Atlantic Oceanographic and Meteorological Laboratory.

Recent updates include improved surface and microphysics schemes and a new shallow convective parameterization scheme. The combination of Geophysical Fluid Dynamics Laboratory (GFDL) surface physics, Slab (thermal diffusion) model, a simplified Arakawa-Schubert scheme for cumulus parameterization, and Ferrier cloud microphysics along with Global Forecast System (GFS) planetary boundary layer scheme is used here. Further details can be found in Tallapragada *et al*.^56^. Osuri *et al*.^57^, affirm the performance capabilities of HWRF in capturing the characteristics of these cyclones.

The HWRF simulations of Phailin and Lehar were performed with initial and boundary conditions from GFS. The model tracks and intensities are compared against observations from Indian Meteorological Division (IMD), and the vortex initialization was provided by Joint Typhoon Warning Center (JTWC) TC Vitals.

Figure 2 Throughout the study, a minimum surface level pressure based center is maintained. The definition of the environmental annulus and the vortex removal (Fig. 2) were done using an adapted version of the Kurihara algorithm^58^, with the notable difference being that we used the model outputs of 10-m winds, as opposed to the 850 mb winds in the original documentation. Here, the environment is defined as the region beyond the radius at which the 10-m wind speed drops below 8 m/s. It is to be noted that as per the definition of the India Meteorological Department, the minimum wind speed that qualifies as a weak depression is 8.75 m/s or 17 knots. Once the boundary between the vortex and the environment was identified, approximately 200 passes of the filter were required to remove the vortex from the environmental field.

Figure 3 The circulation center for tilt computation is calculated using a smoothed, minimum-pressure centroid algorithm. The tilt magnitudes are a strong function of how the circulation center at each level is determined. As explained in Reasor *et al*.^59^ and Ryglicki *et al*.^60^, often, the tilt magnitudes do not correlate well with shear magnitudes. The reader is advised not to take the magnitudes literally. Rather, we intend to focus on the broad takeaways from the trends of the tilt evolution.

Figure 8 The mass flux was computed as a product of density and vertical velocity. The inertial and static stabilities were computed following Vigh and Schubert^54^. The available potential energy and the conversion from potential to kinetic energy are measured by the covariance of heating and temperature, and the covariance of vertical velocity and temperature respectively within the RMW and averaged in the vertical between the surface and 16 km, following Krishnamurti *et al*.^61^.

## Supplementary information

Supplementary Info

## References

[1] Mohanty U (2015). A great escape from the Bay of Bengal “Super Sapphire–Phailin” Tropical cyclone: A case of improved weather forecast and societal response for disaster mitigation. Earth Interactions.

[2] Ooyama KV (1982). Conceptual evolution of the theory and modeling of the tropical cyclone. Journal of the Meteorological Society of Japan. Ser. II.

[3] Marks FD, Shay LK (1998). Landfalling tropical cyclones: Forecast problems and associated research opportunities. Bulletin of the American Meteorological Society.

[4] Hendricks EA, Peng MS, Fu B, Li T (2010). Quantifying environmental control on tropical cyclone intensity change. Monthly Weather Review.

[5] Raymond D, Fuchs Ž, Gjorgjievska S, Sessions S (2015). Balanced dynamics and convection in the tropical troposphere. Journal of Advances in Modeling Earth Systems.

[6] Montgomery MT, Smith RK (2017). Recent developments in the fluid dynamics of tropical cyclones. Annual Review of Fluid Mechanics.

[7] Kaplan J, DeMaria M (2003). Large-scale characteristics of rapidly intensifying tropical cyclones in the North Atlantic basin. Weather and forecasting.

[8] Wood KM, Ritchie EA (2015). A definition for rapid weakening of North Atlantic and Eastern North Pacific tropical cyclones. Geophysical Research Letters.

[9] Kowch R, Emanuel K (2015). Are special processes at work in the rapid intensification of tropical cyclones?. Monthly Weather Review.

[10] Smith RK, Montgomery MT (2015). Toward clarity on understanding tropical cyclone intensification. Journal of the Atmospheric Sciences.

[11] Rogers RF (2016). Observations of the structure and evolution of Hurricane Edouard (2014) during intensity change. Part II: Kinematic structure and the distribution of deep convection. Monthly Weather Review.

[12] Bhatia KT, Nolan DS (2013). Relating the skill of tropical cyclone intensity forecasts to the synoptic environment. Weather and Forecasting.

[13] Tao D, Zhang F (2015). Effects of vertical wind shear on the predictability of tropical cyclones: Practical versus intrinsic limit. Journal of Advances in Modeling Earth Systems.

[14] Jones SC (1995). The evolution of vortices in vertical shear. I: Initially barotropic vortices. Quarterly Journal of the Royal Meteorological Society.

[15] Nguyen LT, Molinari J (2015). Simulation of the downshear reformation of a tropical cyclone. Journal of the Atmospheric Sciences.

[16] Reasor PD, Rogers R, Lorsolo S (2013). Environmental flow impacts on tropical cyclone structure diagnosed from airborne doppler radar composites. Monthly Weather Review.

[17] Corbosiero KL, Molinari J (2002). The effects of vertical wind shear on the distribution of convection in tropical cyclones. Monthly Weather Review.

[18] DeHart JC, Houze Jr RA, Rogers RF (2014). Quadrant distribution of tropical cyclone inner-core kinematics in relation to environmental shear. Journal of the Atmospheric Sciences.

[19] Wadler, J. B., Rogers, R. F. &Reasor, P. D. The relationship between spatial variations in the structure of convective bursts and tropical cyclone intensification as determined by airborne doppler radar. *Monthly Weather Review* (2018).

[20] Corbosiero KL, Molinari J (2003). The relationship between storm motion, vertical wind shear, and convective asymmetries in tropical cyclones. Journal of the Atmospheric Sciences.

[21] Rappin ED, Nolan DS (2012). The effect of vertical shear orientation on tropical cyclogenesis. Quarterly Journal of the Royal Meteorological Society.

[22] Shay LK, Goni GJ, Black PG (2000). Effects of a warm oceanic feature on hurricane opal. Monthly Weather Review.

[23] Jaimes B, Shay LK, Uhlhorn EW (2015). Enthalpy and momentum fluxes during Hurricane Earl relative to underlying ocean features. Monthly Weather Review.

[24] Zhang JA (2017). Observations of infrared sea surface temperature and air–sea interaction in Hurricane Edouard (2014) using gps dropsondes. Journal of Atmospheric and Oceanic Technology.

[25] Wadler, J. B., Zhang, J. A., Jaimes, B. & Shay, L. K. Downdrafts and the evolution of boundary layer thermodynamics in Hurricane Earl (2010) before and during rapid intensification. *Monthly Weather Review* (2018).

[26] Chen H, Gopalakrishnan SG (2015). A study on the asymmetric rapid intensification of hurricane Earl (2010) using the hwrf system. Journal of the Atmospheric Sciences.

[27] Rios-Berrios R, Torn RD (2017). Climatological analysis of tropical cyclone intensity changes under moderate vertical wind shear. Monthly Weather Review.

[28] Smith RK, Zhang JA, Montgomery MT (2017). The dynamics of intensification in a Hurricane Weather Research and Forecasting simulation of hurricane Earl (2010). Quarterly Journal of the Royal Meteorological Society.

[29] Ryglicki, D. R., Doyle, J. D., Jin, Y., Hodyss, D. &Cossuth, J. The unexpected rapid intensification of tropical cyclones in moderate vertical wind shear. Part II: Vortex tilt. *Monthly Weather Review* (2018).

[30] Ryglicki, D. R., Cossuth, J., Hodyss, D. & Doyle, J. D. The unexpected rapid intensification of tropical cyclones in moderate vertical wind shear. Part I: Overview and observations. *Monthly Weather Review* (2018).

[31] Riemer, M., Montgomery, M. T. &Nicholls, M. E. A new paradigm for intensity modification of tropical cyclones: thermodynamic impact of vertical wind shear on the inflow layer. *Atmospheric Chemistry & Physics* **10** (2010).

[32] Riemer M, Montgomery MT (2011). Simple kinematic models for the environmental interaction of tropical cyclones in vertical wind shear. Atmospheric Chemistry and Physics.

[33] Tang B, Emanuel K (2010). Midlevel ventilation’s constraint on tropical cyclone intensity. Journal of the Atmospheric Sciences.

[34] Tang B, Emanuel K (2012). Sensitivity of tropical cyclone intensity to ventilation in an axisymmetric model. Journal of the Atmospheric Sciences.

[35] Riemer M, Laliberté F (2015). Secondary circulation of tropical cyclones in vertical wind shear: Lagrangian diagnostic and pathways of environmental interaction. Journal of the Atmospheric Sciences.

[36] Shenoi S, Shankar D, Shetye S (2002). Differences in heat budgets of the near-surface arabian sea and bay of bengal: Implications for the summer monsoon. Journal of Geophysical Research: Oceans.

[37] Webster PJ, Holland GJ, Curry JA, Chang H-R (2005). Changes in tropical cyclone number, duration, and intensity in a warming environment. Science.

[38] Kotal S, Roy Bhowmik S (2013). Large-scale characteristics of rapidly intensifying tropical cyclones over the bay of bengal and a rapid intensification (ri) index. Mausam.

[39] Van Sang N, Smith RK, Montgomery MT (2008). Tropical-cyclone intensification and predictability in three dimensions. Quarterly Journal of the Royal Meteorological Society.

[40] Krishnamurti, T., Stefanova, L. &Misra, V. Monsoons. In Tropical Meteorology, 75–119 (Springer, 2013).

[41] Pai, D. & Bhan, S. Monsoon 2013: A report (India Meteorological Department, 2013). http://www.tropmet.res.in/kolli/MOL/Monsoon/year2013/Monsoon-2013-NEW.pdf.

[42] Zhang JA, Rogers RF, Reasor PD, Uhlhorn EW, Marks Jr FD (2013). Asymmetric hurricane boundary layer structure from dropsonde composites in relation to the environmental vertical wind shear. Monthly Weather Review.

[43] Brown JM (1979). Mesoscale unsaturated downdrafts driven by rainfall evaporation: A numerical study. Journal of the Atmospheric Sciences.

[44] Molinari J, Frank J, Vollaro D (2013). Convective bursts, downdraft cooling, and boundary layer recovery in a sheared tropical storm. Monthly Weather Review.

[45] Riemer M, Montgomery M, Nicholls M (2013). Further examination of the thermodynamic modification of the inflow layer of tropical cyclones by vertical wind shear. Atmospheric Chemistry & Physics.

[46] Marks Jr FD, Houze Jr RA, Gamache JF (1992). Dual-aircraft investigation of the inner core of Hurricane Norbert. Part I: Kinematic structure. Journal of the Atmospheric Sciences.

[47] Nguyen LT, Rogers RF, Reasor PD (2017). Thermodynamic and kinematic influences on precipitation symmetry in sheared tropical cyclones: Bertha and cristobal (2014). Monthly Weather Review.

[48] Rogers RF, Reasor PD, Zhang JA (2015). Multiscale structure and evolution of hurricane Earl (2010) during rapid intensification. Monthly Weather Review.

[49] Rios-Berrios R, Torn RD, Davis CA (2016). An ensemble approach to investigate tropical cyclone intensification in sheared environments. Part II: Ophelia (2011). Journal of the Atmospheric Sciences.

[50] Leighton H (2018). Azimuthal distribution of deep convection, environmental factors, and tropical cyclone rapid intensification: A perspective from HWRF Ensemble Forecasts of Hurricane Edouard (2014). Journal of the Atmospheric Sciences.

[51] Shapiro LJ, Willoughby HE (1982). The response of balanced hurricanes to local sources of heat and momentum. Journal of the Atmospheric Sciences.

[52] Montgomery MT, Smith RK (2014). Paradigms for tropical cyclone intensification. Australian Meteorological and Oceanographic. Journal.

[53] Stern DP, Vigh JL, Nolan DS, Zhang F (2015). Revisiting the relationship between eyewall contraction and intensification. Journal of the Atmospheric Sciences.

[54] Vigh JL, Schubert WH (2009). Rapid development of the tropical cyclone warm core. Journal of the Atmospheric Sciences.

[55] Miyamoto Y, Takemi T (2015). A triggering mechanism for rapid intensification of tropical cyclones. Journal of the Atmospheric Sciences.

[56] Tallapragada, V. *et al*. Hurricane weather research and forecasting (HWRF) model: 2013 scientific documentation. *HWRF Development Testbed Center Tech. Rep* **99** (2014).

[57] Osuri KK, Nadimpalli R, Mohanty UC, Niyogi D (2017). Prediction of rapid intensification of tropical cyclone Phailin over the Bay of Bengal using the HWRF modelling system. Quarterly Journal of the Royal Meteorological Society.

[58] Kurihara Y, Bender MA, Ross RJ (1993). An initialization scheme of hurricane models by vortex specification. Monthly Weather Review.

[59] Reasor PD, Montgomery MT, Grasso LD (2004). A new look at the problem of tropical cyclones in vertical shear flow: Vortex resiliency. Journal of the Atmospheric Sciences.

[60] Ryglicki DR, Hart RE (2015). An investigation of center-finding techniques for tropical cyclones in mesoscale models. Journal of Applied Meteorology and Climatology.

[61] Krishnamurti T (2005). The hurricane intensity issue. Monthly Weather Review.

