# Author Correction: On the processes influencing rapid intensity changes of tropical cyclones over the Bay of Bengal

**DOI:** 10.1038/s41598-020-77009-x

**Published:** 2020-11-13

**Authors:** Saiprasanth Bhalachandran, R. Nadimpalli, K. K. Osuri, F. D. Marks, S. Gopalakrishnan, S. Subramanian, U. C. Mohanty, D. Niyogi

**Affiliations:** 1 Department of Earth, Atmospheric and Planetary Sciences, Purdue University, West Lafayette, 47906 USA; 2 Indian Institute of Technology Bhubaneshwar, Bhubaneshwar, India; 3 National Institute of Technology, Rourkela, India; 4 NOAA Hurricane Research Division, Miami, FL USA; 5 Department of Agronomy, Purdue University, West Lafayette, 47906 USA

Correction to: *Scientific Reports* 10.1038/s41598-019-40332-z, published online 04 March 2019

This Article contains an incorrect version of Figure 8, where the trend lines did not consider the varying element size in the radial and vertical dimensions during averaging.

Furthermore, in Figure 8, there is an error in the unit for panels (b) and (c).

As a result, Figure 8 legend,

“Time-series plots (averaged in the azimuthal, radial and vertical directions) for Phailin within the RMW. (**a**) Vertical Mass flux in kg/s (**b**) Inertial stability, *I*^2^ (*s*^−1^) (**c**) Static stability, *N*^2^ (**d**) Generation of available potential energy (*m*^2^*s*^−3^) (**e**) Conversion from potential to kinetic energy (*m*^2^*s*^−3^).”

should read:

“Time-series plots (averaged in the azimuthal, radial and vertical directions) for Phailin within the RMW. (**a**) Vertical Mass flux in kg/s (**b**) Inertial stability, *I*^2^ (*s*^−2^) (**c**) Static stability, *N*^2^ (*s*^−2^) (**d**) Generation of available potential energy (*m*^2^*s*^−3^) (**e**) Conversion from potential to kinetic energy (*m*^2^*s*^−3^).”

The correct Figure 8 appears below as Figure 1.

**Figure 1:**
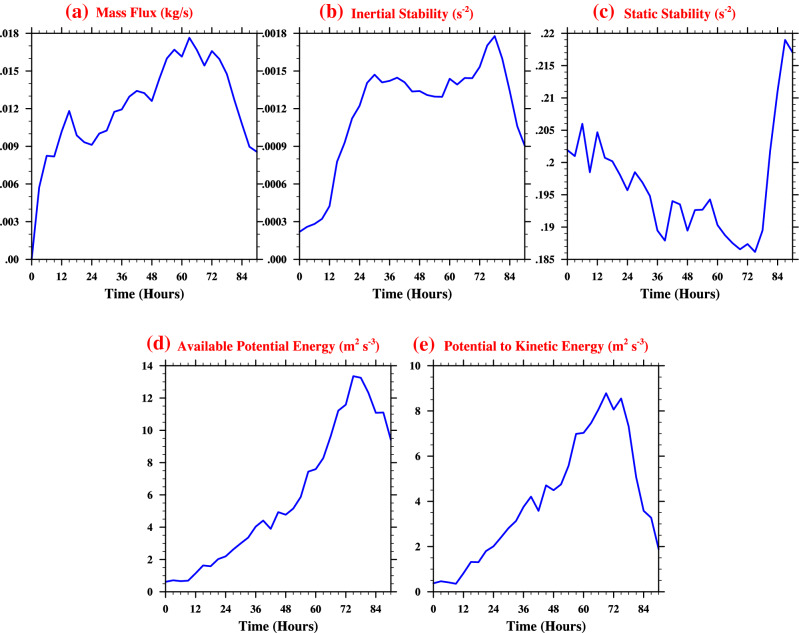
Time-series plots (averaged in the azimuthal, radial and vertical directions) for Phailin within the RMW. (**a**) Vertical Mass flux in kg/s (**b**) Inertial stability, *I*^2^ (*s*^−2^) (**c**) Static stability, *N*^2^ (*s*^−2^) (**d**) Generation of available potential energy (*m*^2^*s*^−3^) (**e**) Conversion from potential to kinetic energy (*m*^2^*s*^−3^).

