# Supplementary material for: On the processes influencing rapid intensity changes of tropical cyclones over the Bay of Bengal
Source: Sci Rep. 2019 Mar 4;9:3382. doi: 10.1038/s41598-019-40332-z (PMC6399276; doi:10.1038/s41598-019-40332-z)
Supplement: Supplementary file 1 — Supplementary Info [file 41598_2019_40332_MOESM1_ESM.pdf]

# On the processes influencing rapid intensity changes of tropical cyclones over the Bay of Bengal

Saiprasanth, B[*1]., Nadimpalli, R[2,1]., Osuri, K. K.[3,1]., Marks Jr, F. D.[4]., Gopalakrishnan, S[4]., Subramanian, S[1].,
Mohanty, U. C.[2], and Niyogi, D.[1,5]

*1. Department of Earth, Atmospheric and Planetary Sciences, Purdue University, West Lafayette, IN, USA*
*2. Indian Institute of Technology Bhubaneshwar, Bhubaneshwar, India*
*3. National Institute of Technology, Rourkela, India*
*4. NOAA Hurricane Research Division, Miami, FL, USA*
*5. Department of Agronomy, Purdue University, West Lafayette, IN, USA*

**Key Words:** Tropical Cyclones, Rapid Intensity Changes, Rapid Intensification, Rapid weakening, Indian Monsoon region, Bay of Bengal, shear-vortex interactions, vertical wind shear, dry-air intrusion, Hurricane Weather and Forecasting Model.

**Nature Scientific Reports**

*Corresponding Author:

Saiprasanth Bhalachandran

sbhalach@purdue.edu

Purdue University

Department of Earth, Atmospheric and Planetary Sciences

West Lafayette, IN 47907

# Supplementary Section

### *Overview of TCs Phailin and Lehar*

TC Phailin originated from a residual cyclonic circulation in the South China Sea on 6th October 2013. Over the next couple of days, winds in association with the cross-equatorial flow generated very intense convection over the Andaman Sea. Satellite observation and buoy data at the time indicated the SSTs to be about ~301-302 K along with an ocean thermal energy of about 600 – 800 $MJ/m^2$. When Phailin underwent rapid intensification beginning at 0600 hours UTC on 10 October 2013, the system had intensified to a Very Severe Cyclonic Storm stage (VSCS) with maximum wind speeds of 115 knots (59 m/s). It is to be noted that India Meteorological Department (IMD) has now refined the VSCS category into two, VSCS (64-89 knots) and extremely VSCS (90-119 knots). A rapid intensification of winds climbing 70 knots from 45 knots to 115 knots, (23.15 m/s to 59 m/s) was observed between 10 and 11 October 2013. The translational speed of the storm was about 4.15 $ms^{-1}$. Phailin made landfall near Gopalpur on the Odisha Coast, India, and the system continued to move north-northwestward. Satellite microwave imagery indicated the eyewall replacement cycle accompanied Phailin's RI as it approached land. Evolved into to the most intense landfalling cyclone since the Odisha Super Cyclone in 1999. Extremely heavy rainfall and flooding in addition to strong gale winds, storm surge and widespread structural damage followed[1].

TC Lehar followed TCs Phailin and Helen during the 2013 season. Just as Helen made landfall on 22 November, Lehar began to develop under very similar circumstances to that of Phailin.

Lehar began as a remnant cyclonic circulation off the South China Sea and began to intensify further as it crossed the coast of Andaman where it led to severe damage. On 25 November 2013, Lehar began moving west-northwestward and intensified in to a VSCS with a maximum wind speed of 75 knots. In contrast to Phailin, Lehar rapidly weakened over the Bay of Bengal from its VSCS stage to a depression as it approached land within only 18 hours of the storm's initial intensification. By the time Lehar reached the coast of Andhra Pradesh at around 0830 UTC on 28th November 2013, the storm was a weak depression and did not cause any notable rainfall or wind damage over the coastal regions.

SUPPLEMENTARY FIGURES

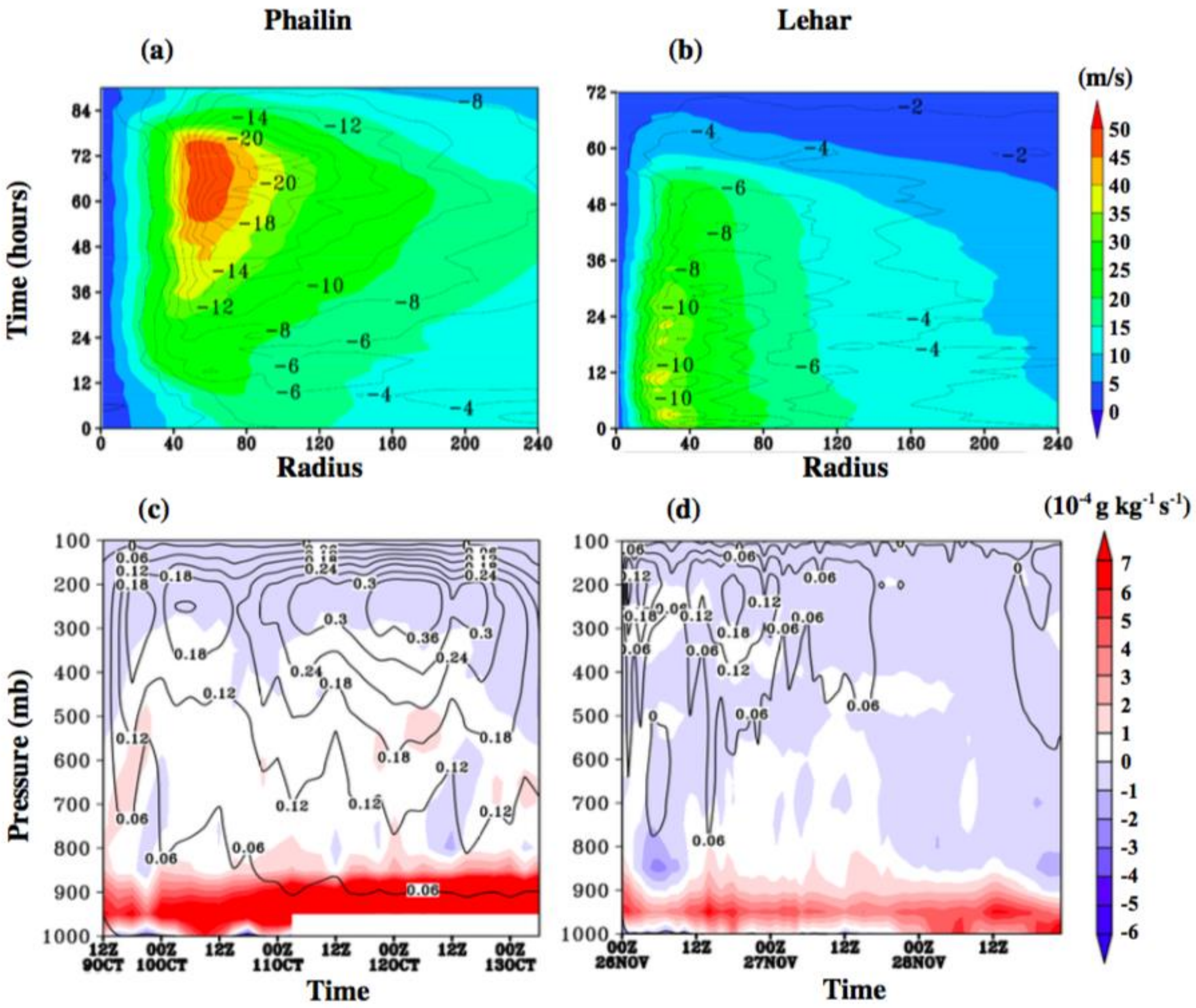


**Supplementary Figure 1:** Contrasting primary and secondary circulations and convective activities in Phailin and Lehar. (a, b) show the Hovmoller plots of azimuthally averaged tangential velocities (shaded) and radial velocities (contours). Time-Height plots of domain averaged (2x2 degrees) horizontal moisture flux convergence (positive values denote convergence) (c,d) with vertical velocity contours.

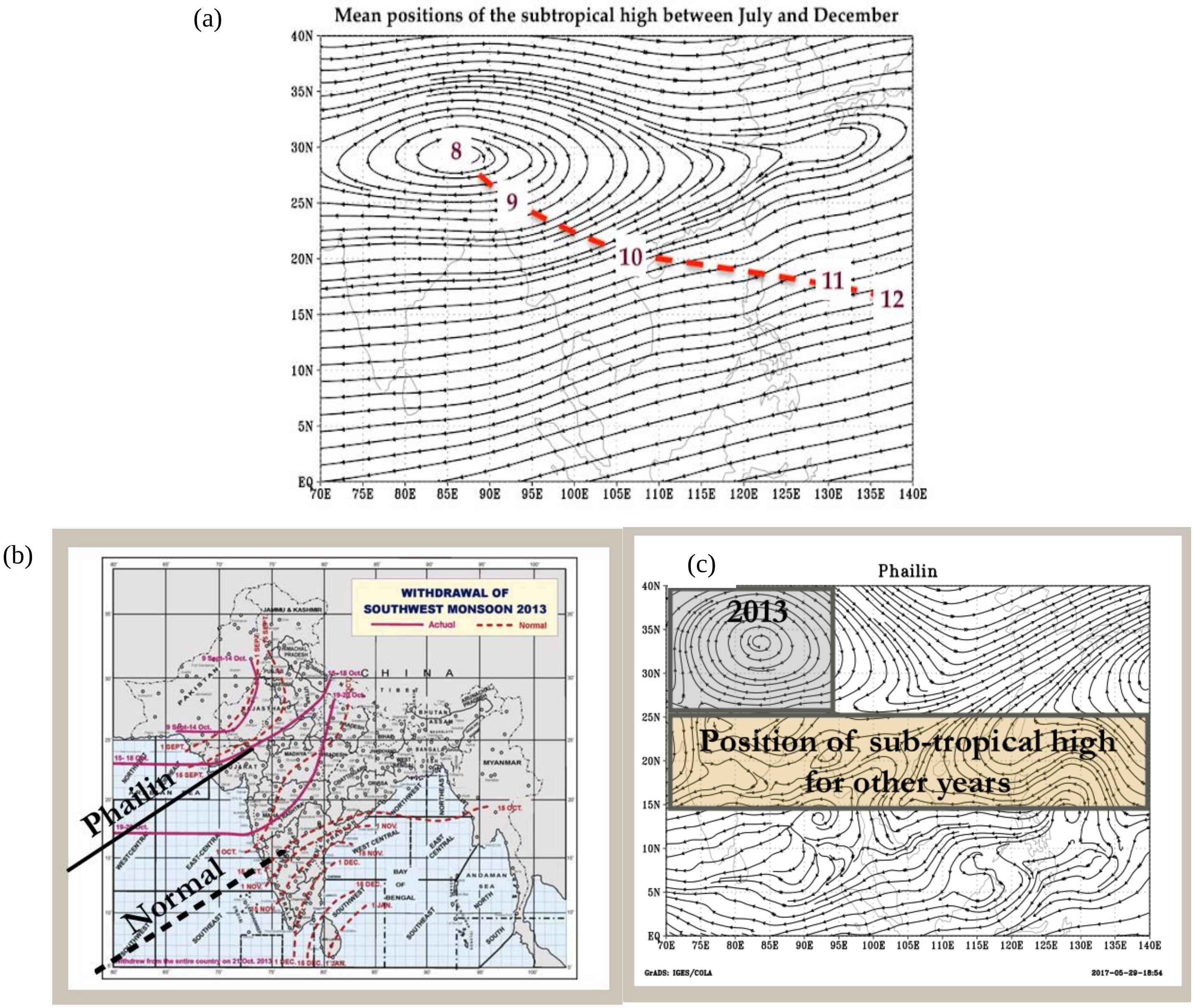


**Supplementary Figure 2**: (a) Mean position of the subtropical high calculated from August to December as per climatology. Computed using ECMWF reanalysis. (b) Withdrawal isochrones of southwest monsoons in the year 2013 highlighting the delay in withdrawal during the lifetime of Phailin. Adapted from the publicly available withdrawal isochrones generated by the India Meteorological Department (c) Streamlines averaged between 200 - 400 mb from GFS indicating the position of the subtropical high during Phailin's time period and the position of the subtropical high for the other between years 1978-2016 computed using reanalysis.

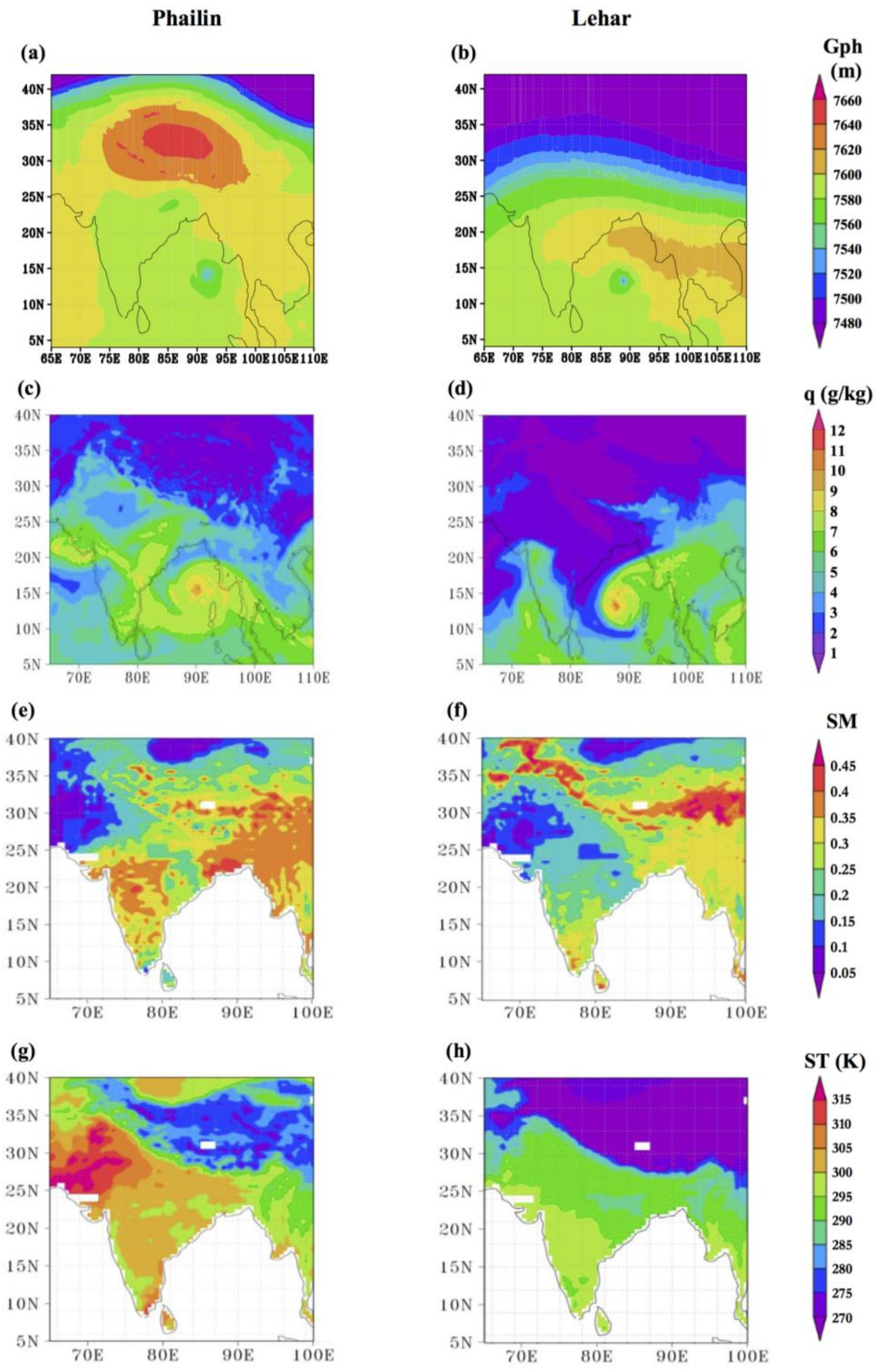


**Supplementary Figure 3:** (a,b) Plot of Geopotential heights at 300 mb for Phailin and Lehar, highlighting the pressure gradient in the meridional direction before the start of weakening and intensification. (c,d) Plots of 500-850 mb integrated specific humidity in g/kg. (e,f,) Plots of antecedent soil moisture (top-layer) in volume fraction (g,h) Plots of antecedent soil temperature (top-layer) (K).

Notes: Suppl. Fig. 3 further serves to explain the differences in the environments of the two TCs, before they diverged to intensify/weaken. Suppl. Figs. 3a and 3b show the geopotential heights of the respective storm environments at 300 mb. In the case of Phailin, the peak geopotential heights are found over the Himalayas, and minimal gradients are found in the proximity of the storm. On the other hand, Lehar's environment is much more baroclinic, with noticeable pressure gradients in the North-south (meridional) direction. These gradients compress the flow field on either side of the ridge (cf. Figure 2b) and drive the shear experienced by Lehar. Suppl. Figs. 3c and 3d show the vertically integrated specific humidity in the storm environments. Suppl. Fig. 3c indicates that the cold, dry continental air was restricted to the Himalayan region and the environment in the vicinity of Phailin was moisture-rich. However, over the next six weeks, the cold and dry, continental air was drawn south into the flow field of the ridge and Lehar. In addition to the seasonality, we speculate that there is a possibility that Lehar's two preceding TCs - Phailin, and Helen, drew a lot of moisture from their environments and altered the synoptic environments for Lehar (not explored in this study). Due to the delayed withdrawal of monsoons at the time of Phailin, the antecedent soil moisture was higher, and the soil was warmer (Suppl. Fig. 3e, 3g). In contrast, due to the onset of winter by late November, Lehar's antecedent soil temperatures were colder (Suppl. Fig. 3f) and despite rainfall from TC Helen (landfall location 15N, 80E degrees) during the same time that Lehar was traversing across the Bay of Bengal, the net soil moisture content was markedly lower than Phailin's (Suppl. Fig. 3h).

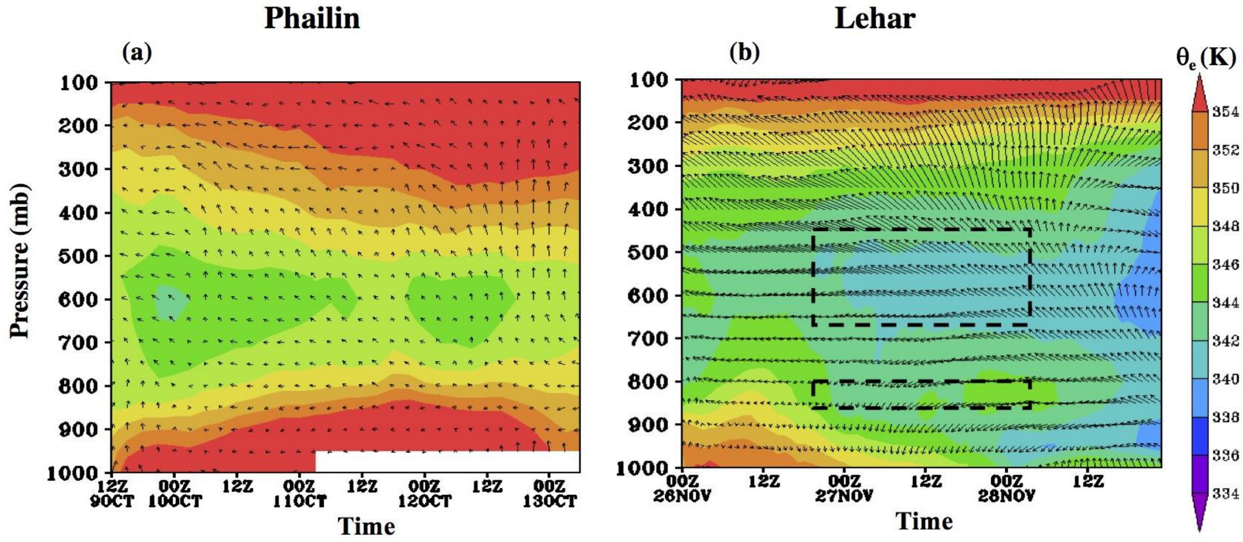


**Supplementary Figure 4**: Time-Height plots of the (3°x 3°) domain averaged $\boldsymbol{\theta}_e$. These plots provide an at-a-glance, spatial averaged viewpoint.

(a)

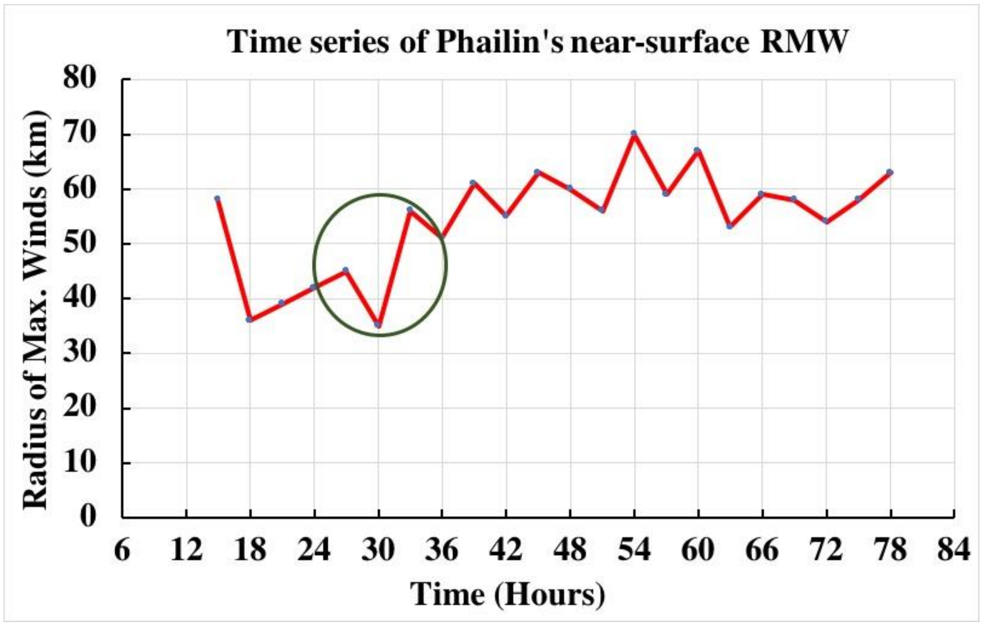


(b)

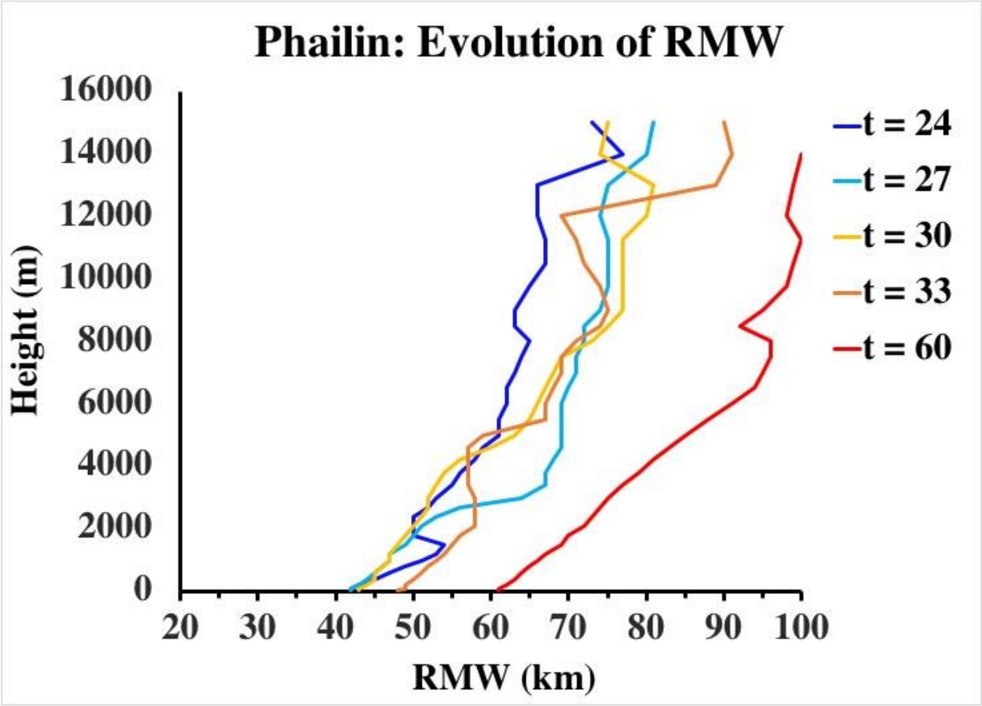


**Supplementary Figure 5**: (5a) Time series of the 10-m radius of maximum winds (RMW) for Phailin. Highlighted are times 27 to 33, where there is an eyewall replacement cycle. (5b) RMW computed at each height at various times as the storm intensifies. These figures serve to illustrate the expansion of RMW during the intensification of Phailin.

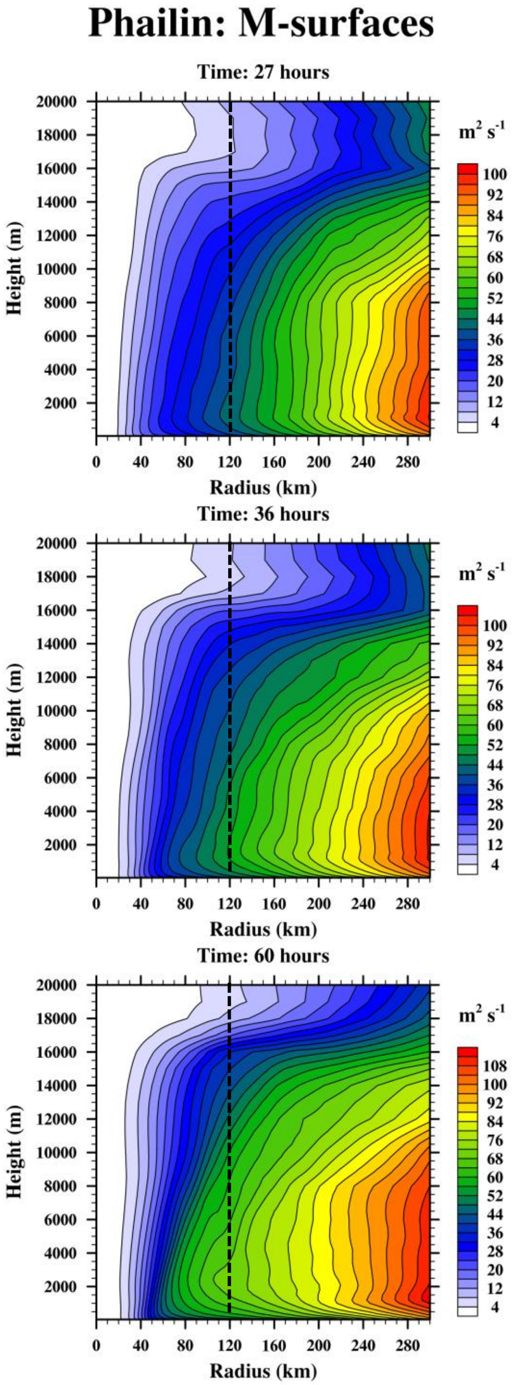


**Supplementary Figure 6:** Inward motion of Phailin's angular momentum surfaces as the storm intensifies. Radius-Height plots of angular momentum at times t = 27, t = 36, t = 60 hours. The dashed line is simply plotted for reference to show the inward motion.

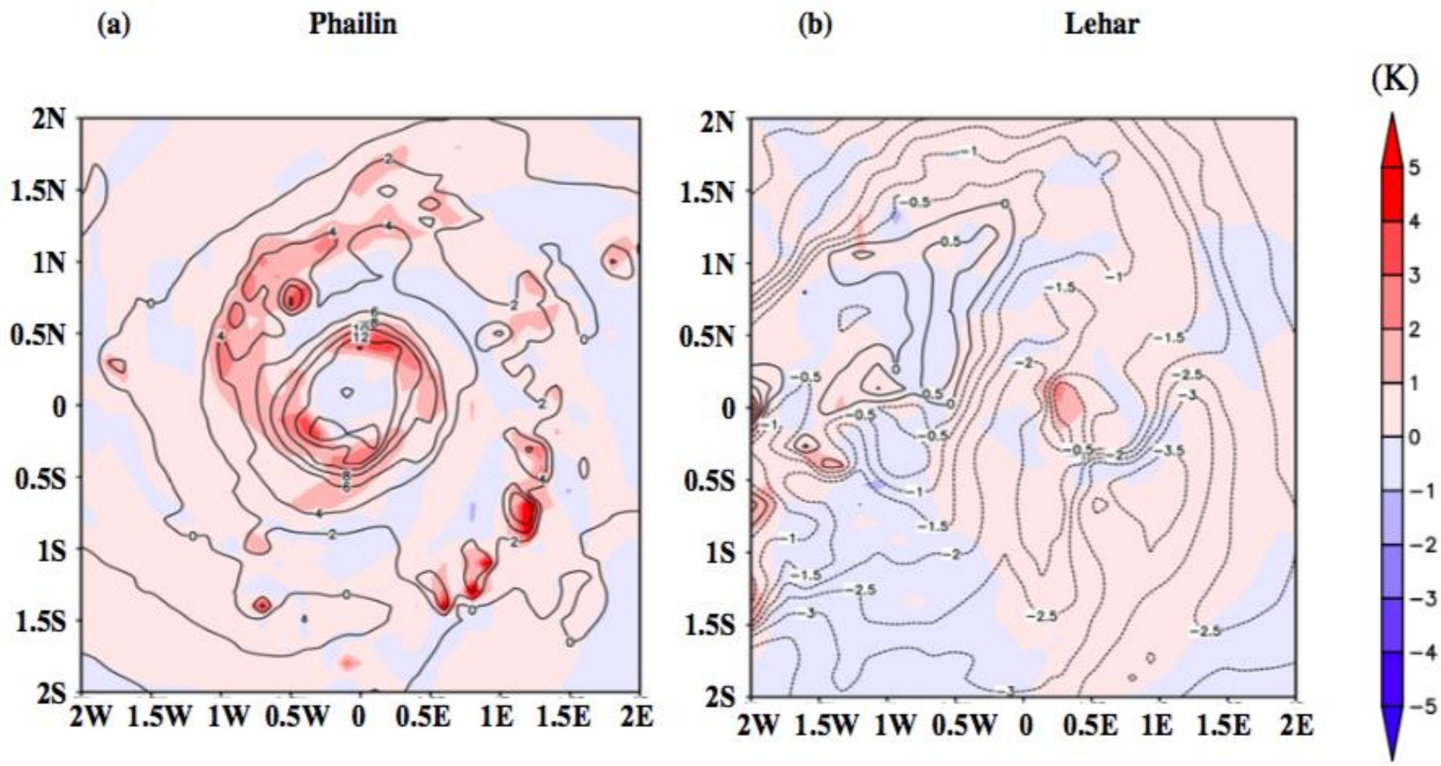


**Supplementary Figure 7**: Plot of vertical velocities (shaded, in m/s) and equivalent potential temperature anomalies (contours, in K) highlighting the updrafts and downdrafts at the start of intensification (t = 33 hours) for Phailin and at the start of weakening (t = 27) for Lehar. The intense red spots indicate the vortex-scale deep convective updrafts. The moderate red regions indicate the moderate, mesoscale upward motion. The moderately blue regions indicate the downward motion. This figure serves to illustrate the importance of the upper level configuration for the wrapping of convection. While the upper level configuration was conducive for the wrapping of convection in Phailin (a), the destruction of the upper level features due to shear in Lehar (b) do not allow for the development of these pockets of convection.
